# 3D spheroids of human placenta-derived mesenchymal stem cells attenuate spinal cord injury in mice

**DOI:** 10.1038/s41419-021-04398-w

**Published:** 2021-11-22

**Authors:** Junhao Deng, Miao Li, Fanqi Meng, Zhongyang Liu, Song Wang, Yuan Zhang, Ming Li, Zhirui Li, Licheng Zhang, Peifu Tang

**Affiliations:** 1 Medical School of Chinese PLA, Beijing, 100853 China; 2 Department of Orthopedics, The Chinese PLA General Hospital, Beijing, 100853 China; 3 Key Laboratory of Chemical Genomics, Shenzhen Graduate School, Peking University, Shenzhen, 518055 China; 4 Department of Spine Surgery, Peking University People’s hospital, Beijing, 100044 China; 5 Medical college, Nankai University, Tianjin, 300071 China; 6 IBM Research-China, Beijing, 100193 China; 7 Present Address: Department of Anesthesiology, Columbia University, New York, NY 10032 USA

**Keywords:** Spinal cord injury, Mesenchymal stem cells

## Abstract

Mesenchymal stem cell (MSC) is an absorbing candidate for cell therapy in treating spinal cord injury (SCI) due to its great potential for multiple cell differentiation, mighty paracrine secretion as well as vigorous immunomodulatory effect, of which are beneficial to the improvement of functional recovery post SCI. However, the therapeutic effects of MSC on SCI have been limited because of the gradual loss of MSC stemness in the process of expanding culture. Therefore, in this study, we aimed to maintain those beneficial properties of MSC via three-dimensional spheroid cell culture and then compared them with conventionally-cultured MSCs in the treatment of SCI both in vitro and in vivo with the aid of two-photon microscope. We found that 3D human placenta-derived MSCs (3D-HPMSCs) demonstrated a significant increase in secretion of anti-inflammatory factors and trophic factors like VEGF, PDGF, FGF via QPCR and Bio-Plex assays, and showed great potentials on angiogenesis and neurite morphogenesis when co-cultured with HUVECs or DRGs in vitro. After transplantation into the injured spinal cord, 3D-HPMSCs managed to survive for the entire experiment and retained their advantageous properties in secretion, and exhibited remarkable effects on neuroprotection by minimizing the lesion cavity, inhibiting the inflammation and astrogliosis, and promoting angiogenesis. Further investigation of axonal dieback via two-photon microscope indicated that 3D-HPMSCs could effectively alleviate axonal dieback post injury. Further, mice only treated with 3D-HPMSCs obtained substantial improvement of functional recovery on electrophysiology, BMS score, and Catwalk analysis. RNA sequencing suggested that the 3D-HPMSCs structure organization-related gene was significantly changed, which was likely to potentiate the angiogenesis and inflammation regulation after SCI. These results suggest that 3D-HPMSCs may hold great potential for the treatment of SCI.

## Introduction

Spinal cord injury (SCI) is a debilitating neurological disease worldwide, which leads to the loss of motor as well as sensory neuronal function and ultimately causes severe psychological, physical, and social dysfunction on suffered patients [1, 2]. Epidemiological data showed that the mortality of acute SCI ranges from 4.4 to 16.7% in hospital in the world [3]. However, there is currently no clinically-effective strategy in the treatment of SCI, as existing treatments like surgical decompression [4], high-dose methylprednisolone [5, 6], or neurotrophic factors protection [7] rely heavily on the patient’s limited capacity to self-repair and rebuild the disrupted network [8].

Recent studies [8, 9] have shown that stem cells therapy held great promise for SCI treatment. A wide range of stem cells have been used as candidates for transplantation in brain and spinal cord injuries [10–13]. Among these various types of cells, human placenta-derived mesenchymal stem cells (HPMSCs) might be of great potential for the treatment of SCI. Like MSCs, HPMSCs are of favorable immunomodulatory, neuroprotective and neuroregenerative properties as they are able to secrete varieties of bioactive molecules beneficial for neural regeneration, and they, though controversial, might be capable of lesion-directional migration and differentiation into neuron- or astrocyte-like cells [14, 15]. Moreover, HPMSCs are partially characterized by primitive embryonic feature. They demonstrate higher potential to differentiate into many cell types, greater proliferative ability, stronger immunomodulatory capacity, and lower immunogenicity when compared with adult MSCs [15–17]. Besides, HPMSCs can be easily harvested from placenta as they are usually regarded as discarded waste at birth, which effectively avoid ethnic conflicts. In addition, HPMSCs were reported to be of good neuroprotective properties [18], making these cells attractive in neural tissue repair. However, it is currently lacking researches on HPMSCs in the treatment of SCI.

On the other hand, numerous studies have reported that a sufficient number of transplanted cells in the injured site is a prerequisite for tissue repair [19–21]. However, injury-induced hostile microenvironment would largely reduce the number of transplanted cells around the lesion after transplantation. Therefore, more and more strategies have been introduced to improve the survival and viability of HPMSCs after implantation, such as incorporation cells into biomaterials [22–25], neurotrophic factor gene modification of cells [26–29], and application of suitable stimuli [30], etc. Though these strategies showed some good pathological and functional outcomes in the treatment of SCI, they were challenged by many side effects they might cause in the treatment process. For example, the degradation products of biomaterials may have side effects on the recipient’s system [31]. Virus-based transfection method used to increase the expression of neurotrophic factors may change the recipient’s innate fate and cause unpredictable results, and physical irritations (like electricity, magnetic exposure, and et al.) often need to be applied via external large-sized devices which may be inconvenient to put into practice in clinic [32]. Therefore, a simple, easy-to-use, and high-efficiency strategy should be developed to make HPMSCs available for SCI repair. Evidence have shown that 3D cultivation of donor cells in vitro before transplantation exhibit excellent properties in the treatment of central nervous system diseases, such as Alzheimer’s disease [33], spinal cord injury [34, 35], and brain trauma [36]. However, the underlying mechanism by which 3D cultivation improves SCI repair remains unknown. Therefore, it is urgent to evaluate the effect of 3D-HPMSCs cultivation on SCI treatment and explore the potential mechanism behind them.

In the present study, we first established the 3D-HPMSCs cultivation system and characterized its properties by comparing them with the 2D cell sheet, and further evaluated the therapeutic effects of 3D-HPMSCs on SCI. We proved that 3D-HPMSCs could survive in the damaged spinal cord, efficiently modulate the exaggerated inflammation and astrogliosis, and better improve functional recovery of SCI mice, which suggested 3D-HPMSCs might be a potentially practical therapy for spinal cord injury.

## Methods and materials

### Isolation, culture, and fluorescent label of MSCs

With a view to meeting the clinical need, we took advantage of human MSCs which were isolated from human placenta as described previously [37]. In brief, full-term (38–40 weeks gestation) placentas were obtained from healthy donors with written informed consent and the procedure was approved by the Ethics Committee of PLA General Hospital. The tissue was washed several times with cold phosphate-buffered saline (PBS) before it was mechanically minced and enzymatically digested with 0.25% trypsin-ethylene diamine tetraacetic acid (EDTA; Gibco-Invitrogen) for thirty minutes at 37 °C in a water bath. Then the digest was filtered, pelleted, and resuspended in a growth medium which consists of Dulbecco’s modified Eagle’s medium (DMEM; Gibco-Invitrogen), 10% fetal bovine serum (FBS; Gibco-Invitrogen) and antibiotics. Cells were subsequently seeded on uncoated polystyrene dishes and medium would be replaced every 2 days to reach 80% confluence. Cells were sub-cultured after trypsinization.

In order to track the transplanted cells in mice, we used concentrated GFP-lentivirus (BrainVTA, Wuhan, China) to infect the target cells. After cells culture in the medium with lentivirus for 16 h, the fresh medium was added and continue the culture for another 8 h. Then the GFP-labeled MSCs were selected by the Fluorescence-activated cell sorting.

### Spheroid culture

To form spheroids, passage 3–5 MSCs were chosen to be cultured by hanging-drop method as described previously with some modifications [38, 39]. Briefly, cells were seeded in hanging drop within DMEM containing 10% FBS and 2 × 10^4^ cells per drop and incubated for 36 h. The spheroids were then transferred to a suspension culture and incubated in new growth medium for another 24 h.

### Cell morphology and phenotype characterization

Cells morphology was first captured by microscope, and then the cell size of both types was also measured. Specifically, single cells of 2D-MSCs and 3D-MSCs were transferred to the chambers of a hemocytometer orderly, and they were carefully observed under the optical microscope. Pictures were obtained by using a Ds-Fil camera.

For the phenotype characterization, MSC specific surface marker expression was assessed by flow cytometry. HPMSCs were isolated and then washed with Phosphate Buffer Saline (PBS). Next, cells were incubated with surface antibodies against CD34-PE, CD45-PerCP, CD73-APC, CD90-FITC, and CD105-V450 (BD Biosciences Pharmingen, San Jose, CA, USA) for 30 mins under room temperature.

### Cells proliferation and apoptosis

For cells proliferation analysis, cell counting kit—CCK8 (Hanbio, Shanghai, China) was applied to test cell proliferation according to the manufacturer’s instructions. Briefly, MSCs of both types were seeded in 96-well plates with a density of 5*10^3^ cells per well and tested at 0 days, 1 days, 2 days, 3 days, and 5 days post culture. The culture medium would be replaced with 100 ul medium and 10 ul CCK8 at each observation time point. The absorbing result at 450 nm was recorded by the microplate reader (Bio-Rad, USA).

For cells apoptosis analysis, flow cytometry was again used to determine whether 3D culture would affect cells survival. About 2 × 10^5^ cells were stained with the Annexin V-PE and FITC apoptosis detection kit (BD Biosciences Pharmingen, San Jose, CA, USA) in strict accordance with the supplier’s instruction. The same procedure would be repeated for twice.

### HUVECs or DRG co-culture with HPMSCs

Human Umbilical Vein Endothelial Cells (HUVECs) were gifts from Prof. Yu Wang (Orthopedics Institute of Chinese PLA, Chinese PLA General Hospital, Beijing, China). HUVECS were cultured and expanded in DMEM supplemented with 10% human serum (Gibco, Carlsbad, CA, USA) and 1% Penicillin&Streptomycin solution (Gibco, Carlsbad, CA, USA). HPMSCs of appropriate passage number were resuspended in the same medium and then added to HUVECs at a ratio of 1:1 (the cell density of 1*10^4^/ml each) in u-Slide Angiogenesis (Ibidi, Verona, Germany), allowing direct cell-to-cell physical contact and paracrine communication. Then All-in-one Fluorescence microscopic imaging system (Keyence, Osaka, Japan) was used to real-time image the co-culture system for consecutive 5 hours. Five fields were randomly selected under a 100X microscope to observe and count the number of tubes formed by vascular endothelial cells. In the co-culture system, HPMSCs was at passage 4, and HUVECs at passage 4.

For dorsal root ganglion (DRG) culture, spinal cords were carefully isolated from newborn C57BL/6 mice and the DRG were carefully excised and seeded on the Polylysine-coated 6-well plate [40]. Subsequently, MSCs at the same cell density of 5*10^5^/ml, 3D-MSCs or 2D-MSCs, were added to the 6-well plate immediately. After 7 days co-culture, the co-culture system was fixed with 4% paraformaldehyde (PFA) and prepared for immunofluorescence staining of primary antibody NF200 and S100, and secondary antibody Alexa fluor 488 and 594 (Abcam, Cambridge, UK). The neurite growth was analyzed using Sholl analysis as previously stated [41].

### Real-time PCR analysis

Total RNAs in each group were isolated and extracted using TRIzol reagents following the manufacturer’s protocol. First-strand cDNA was prepared by reverse transcription with Superscript II reverse transcriptase and oligo(dT) primers and stored at −20 °C. Real-time PCR (RT-PCR) was performed using with SYBR Green Real-Time PCR Master Mix on an ABI 7300 QPCR System. As an internal control, we also quantified the levels of glyceraldehyde-3-phosphate dehydrogenase (GAPDH) in comparison with the targeted genes. Data were collected, normalized, and analyzed using 2^−△△Ct^ method. The designed primers were listed in Table 1.

**Table 1:** The prime sequences for RT-PCR.

*Genes (for mRNA)*	*Sequence (5'-3')*
*VEGF*	*Forward: AGGAGGAGGGCAGAATCATCA* *Reverse: CTCGATTGGATGGCAGTAGCT*
*NT-3*	*Forward: CATCCCAAACCTACGTCCGAG* *Reverse: TCTCGACAAGGCACACACACAG*
*IL-4*	*Forward: TCATTTTCCCTCGGTTTCAG* *Reverse: AGAACAGAGGGGGAAGCAGT*
*IL-10*	*Forward: TCAGGGTGGCGACTCTAT* *Reverse: TGGGCTTCTTCTAAATCGTTC*
*IL-11*	*Forward: TCTCTCCTGGCGGACACG* *Reverse: AATCCAGGTTGTGGTCCCC*
*IL-13*	*Forward: GTCAGGCTGCAGTGCCATCG* *Reverse: TTGAACCGTCCCTCGCGAAA*
*GAPDH*	*Forward: AGGTCGGTGTGAACGGATTTG* *Reverse: TGTAGACCATGTAGTTGAGGTCA*

Note: BDNF brain-derived neurotrophic factor, VEGF, vascular endothelial growth factor, NT-3 neurotrophin-3, IL interleukin, GAPDH glyceraldehyde-3-phosphate dehydrogenase.

### Enzyme-linked immunosorbent assay (ELISA)

IL-4, IL-10, IL-13, TNF-α, and IL-6 ELISA kit were purchased from Jiangsu Meimian industrial Co., Ltd, China. Cells were cultured for 24 h in DMEM with 10% FBS, and the supernatant of both cell types was then collected to measure the levels of IL-4, IL-10, IL-13, TNF-α, and IL-6 according to the manufacturer’s instruction. As for tissue sample, 1 cm-long spinal cord (cells grafts were at the center of the spinal cord) was lysed with RIPA Lysis Buffer and then quantified with Pierce bicinchoninic acid (BCA) protein assay kit (Beyotime, Shanghai, China). Thereafter, the tissue supernatant was prepared for ELISA using their respective kits according to the manufacturer instructions.

### Human cytokine, chemokine, and trophic factors Bio-Plex Pro^TM^ assays

Cells and tissue samples were assayed using and Bio-Plex Pro^TM^ human cytokine 27-plex assay kit (Bio-Rad#10014905) according to the manufacturer’s instructions. As for cells sample, cell supernatant from 3D- and 2D-HPMSCs at a density of 10^5^/ml were collected after appropriate culture. As for tissue sample, 1cm-long spinal cord (cells grafts were at the center of the spinal cord) was lysed with RIPA Lysis Buffer and then quantified with Pierce bicinchoninic acid (BCA) protein assay kit (Beyotime, Shanghai, China). All the cells and tissue supernatant were next prepared for assay. Data was processed using Bio-Plex Manager software version 6.1 (Bio-Rad Laboratories, USA).

## RNA-sequencing analysis

### Sample collection, preparation, and sequencing

RNA extraction procedure was performed as mentioned above. RNA integrity was evaluated using the RNA Nano 6000 Assay Kit of the Bioanalyzer 2100 system (Agilent Technologies, CA, USA). Then, they would be prepared for cDNA amplification, library construction, and quality control. Once the quality of cDNA library was confirmed, the clustering of the index-coded samples was performed on a cBot Cluster Generation System using TruSeq PE Cluster Kit v3-cBot-HS (Illumia) according to the manufacturer’s instructions (from Beijing Novel Bioinformatics Co., Ltd. (https://en.novogene.com/)). After cluster generation, the library preparations were sequenced on an Illumina Novaseq platform and 150 bp paired-end reads were generated.

### Data process

Raw data (raw reads) of fastq format were firstly processed through in-house Perl scripts. In this step, clean data (clean reads) were obtained by removing reads containing adapter, reads containing N base, and low-quality reads from raw data. At the same time, Q30 and GC content of the clean data were calculated. All the downstream analyses were based on the clean data with high quality.

Reference genome and gene model annotation files were downloaded from genome website directly. Index of the reference genome was built using Hisat2 v2.0.5 and paired-end clean reads were aligned to the reference genome using Hisat2 v2.0.5. We selected Hisat2 as the mapping tool for that Hisat2 can generate a database of splice junctions based on the gene model annotation file and thus a better mapping result than other non-splice mapping tools.

As for the quantification of gene expression level, FeatureCounts v1.5.0-p3 was used to count the reads numbers mapped to each gene. And then FPKM of each gene was calculated based on the length of the gene and reads count mapped to this gene. FPKM, expected number of Fragments Per Kilobase of transcript sequence per Millions base pairs sequenced, considers the effect of sequencing depth and gene length for the reads count at the same time, and is currently the most commonly used method for estimating gene expression levels.

### Data analysis

Prior to differential gene expression analysis, for each sequenced library, the read counts were adjusted by edgeR program package through one scaling normalized factor. Differential expression analysis of two conditions was performed using the edgeR R package (3.22.5). The P values were adjusted using the Benjamini & Hochberg method. Corrected *P*-value of 0.05 and absolute foldchange of two were set as the threshold for significantly differential expression.

As for the enrichment analysis of differentially expressed genes, both Gene Ontology (GO) and KEGG enrichment analysis were implemented by the clusterProfiler R package in this study, in which gene length bias was corrected. Corrected *P*-value less than 0.05 were considered significantly enriched by differential expressed genes.

To explore the potential interactive relationships among the DEGs, Protein-protein interaction (PPI) analysis was also performed based on the STRING database (https://string-db.org/cgi/input.pl), and the PPI network was visualized by the Cytoscape 3.8.2 software.

The RNA-seq data in this study was submitted to Gene Expression Omnibus (GEO) (http://www.ncbi.nlm.nih.gov/geo/) under accession ID GSE174619.

### Animals

Wild-type C57BL/6 mice were purchased from Guangdong Medical Laboratory Animal Center, and Thy1-YFP transgenic mice (H-line, certification No. 2013–0002) were obtained from Jackson Laboratory and bred in the Experimental Animal Centre of Shenzhen Graduate School of Peking University, which can specifically express a high level of yellow fluorescent protein (YFP) in motor and sensory neurons, dendrites, and axons. Female mice (5–6 weeks of age, 14–18 g) were used for all experiments. To be specific, C57 mice and YFP H-line mice were equally and randomly divided into three groups after SCI (the PBS, 2D-HPMSC-treated, and 3D-HPMSC-treated groups) (Table 2). The number of animals used in each group was based on our previous studies [42]. All animal experiments were minimized the number of animals but ensured adequate power to detect the difference between different group. All animal surgical procedures were approved by the Committee of the Animal Experimentation Ethics of the Chinese PLA General Hospital.

**Table 2:** Experimental groups.

Time points	Analysis	Groups		
		PBS	2D MSC	3D MSC
3 days	Cells survival	-	6	6
	GFAP/Iba-1 fluorescence	6	6	6
	In vivo imaging	6^*^	6^*^	6^*^
7 days	Cells survival	-	6	6
	GFAP/Iba-1 fluorescence	6	6	6
	In vivo imaging	6^*^	6^*^	6^*^
	Functional evaluation	6^#^	6^#^	6^#^
28 days	Cells survival	-	6	6
	GFAP/Iba-1 fluorescence	6	6	6
	PECAM fluorescence	6	6	6
	In vivo imaging	6^*^	6^*^	6^*^
	Electrophysiology assessment	3	3	3
	Functional evaluation	6^#^	6^#^	6^#^
	Total	39	57	57

The number of C57 mouse used for each group at each point.

* and ^#^ means the number of YPF transgenic mouse and C57BL/6 mouse, respectively. These mice were repeatedly used at each time point and not required to be executed until the last time point.

### SCI model and cell transplantation

The thoracic spinal cord hemisection procedure was performed as previously described [43–45]. In brief, all the mice were anesthetized intraperitoneally with sodium pentobarbital at 80 mg/kg in 0.9% NaCl. Then the dorsal surface above the thoracic spinal region of each mouse were shaved and washed with iodophor for twice to prevent the infection. Then the mice underwent a laminectomy at the level of T10 spinal cord. A sharp scalpel and an iridectomy scissor were used to transect the whole right spinal cord from the middle of the spinal cord to the lateral side. If the mouse was not paralyzed in the injured side, it would be excluded for further analysis. For in vivo imaging, a model of pinprick injury in the mouse spinal cord were used to avoid the influence of blood as described previously [46]. Briefly, all procedures were similar to the process of the hemisection injury except the area of the lesion. We stabbed the spinal cord tissue just next to the dorsal central vein by a sharp pin to induce the spinal cord injury at the same level. All operations were performed under a stereomicroscope.

Then all mice were prepared for transplantation immediately. As for transplantation experiments [47], 2D and 3D-HPMSCs were harvested and were subsequently washed twice with PBS, resuspended in PBS, and kept on ice until transplantation. These animals from different groups received corresponding transplantation of PBS (control), 2D-HPMSCs, or 3D-HPMSCs. A motorized stereotaxic injector pump (RWD Life Science, China) with a 34 gauge needle attached to a 10 μl Hamilton Syringe was put right above the lesion site. Then the needle was stereotactically inserted into the spinal cord at a depth of 1 mm and 1 × 10^5^ cells in 2 μl were injected over at least 4 min. In order to prevent the backflow of the injection, the needle was then kept in situ for an extra four minutes. After that, for transplantation performed on C57 mice, we sutured the back muscle and skin, and then put them into warm cages. Bladders were manually emptied each day until animals were able to urinate independently.

### In vivo imaging by two-photo microscope

For transplantation carried on YFP H-line mice, a self-designed spinal stabilization device was used for in vivo imaging as described previously [48, 49] with minor modifications. Briefly, we used to metal bars to clamp three of vertebrates on both sides of the spinal cord lesion area. After the immobilization of the spinal cord, we carefully cleared the blood around the lesion site with sterile PBS to avoid the influence of blood in the imaging process. Upon having achieved a clean surface of the spinal cord, we used a 2% agarose to cover the lesion site and its surroundings, and waited for agarose’s solidification. Then a sharp scalpel was used to cut the center part of the agarose so that a small pool was produced to reserve water for imaging. We used an Olympus Fluo View FV1000 two-photon microscope with an Olympus 10 × 1.0NA water-immersion objective lens and turned the laser to 920 nm for the imaging of axons as well as the graft of cells. For long-term repeated imaging, we used some obvious features of axons and transplanted cells to help navigate the same region of interest. Fifteen to twenty axons were measured for each animal [49].

### Image processing and quantification

NIH Image J software was used to analyze the in vivo image captured by two-photon microscope. In an effort to increase the clarity of images, we slightly adjusted the brightness and contrast. Then we manually tracked the dieback of individual axons both from the rostral and the caudal area in a Z-stack image to evaluate the distance of each axon tips from the edge of observed lesion site. We measured 15–20 axons for each mouse to determine the average dieback distance. The measurements of all mice in each group were collected to yield the average dieback distance per time point.

### Histology and Immunofluorescence stain

After the survival time of 3, 7, and 28 days, animals were deeply anesthetized and transcardially perfused with 0.01 mol/L PBS, as well as a fixative (4% paraformaldehyde in 0.01 mol/L PBS). Subsequently, the 1-centimeter-long spinal cord tissue containing the lesion was removed and immersed in the same fixative at 4 °C overnight, followed by 20% sucrose at 4 °C for 1 day and 30% sucrose at 4 °C for another. After that, samples were embedded in optical cutting temperature compound (OCT) and were then sectioned on a cryostat at 40 um thickness. To determine the lesion size, inflammatory infiltrate, and gliosis, spinal cord sections were washed by PBS for three consecutive times and were pre-incubated by 10% goat serum in PBS containing 0.5% Triton X-100 for no less than 1 h at room temperature. Then the primary antibodies were incubated overnight at 4 °C, including rat anti-glial fibrillary acidic protein (GFAP) (Thermo Fisher Scientific, USA; 13-0300), rabbit anti-ionized calcium-binding adapter molecule 1 (Iba-1) (Wako, Japan; 019-19741), rabbit anti-neurofilament (Sigma-Aldrich, USA; N4142), and mouse anti- CD31/PECAM-1 antibody (SCBT, USA; sc-376764). In the next day, we repeated the washing steps, and the following secondary antibodies were applied for 1 h at room temperature: goat anti-rat Alexa Fluor 647 (Abcam, UK; ab150167), goat anti-rabbit Alexa fluor 555 (Abcam, UK; ab150074), donkey anti-rabbit Alexa fluor 488 (Abcam, UK; ab150077). And sections were also mounted by fluoromount G (Sigma-Aldrich, USA; 81381). An Olympus fluorescence microscope was used to perform immunofluorescence analysis. In order to improve the comparability and readability, the contrast and brightness of images was regulated equally in each group.

### Quantification of stained tissue sections

To measure the lesion size in immunostained sections, 5 to 7 images per animal comprising the lesion site obtained by the fluorescence microscope were manually outlined and quantified by Image J software, as previously described [45, 47]. In brief, lesion size was determined by anti-GFAP immunofluorescence. For quantification of gliosis and inflammatory infiltrate, 5 to 7 images per animal were analyzed. Specifically, intensity analysis was applied to the measurements of gliosis (GFAP expression) and microglial activation (Iba-1 expression). The intensity analysis was performed within square areas measuring 100 × 100μm extending 600 μm both rostrally and caudally from the lesion center.

## Behavioral analysis

### BMS score

All mice received locomotion tests as soon as they had become totally conscious after surgery. The functional recovery in SCI mice was measured for four consecutive weeks by virtue of the Basso Mouse Scale (BMS) [50]. The BMS is a 10-point locomotor rating scale, in which 9 point means normal locomotion while 0 equals to complete paralysis. Two investigators, who are blinded to the experiment, independently gave the score of the mice. The scores are based on the hind limb movement of mice observed in an open field for at least 4 minutes.

### Catwalk XT gait analysis

Mice gaits were analyzed using a video-based analysis system—Catwalk XT, Noldus, Wageningen, Netherlands. Briefly, mice were required to cross a walkway with an illuminated glass wall, and real-time images would be captured when mice’s toes contacted the glass floor. On the basis of toes position, pressure, and surface area, multiple parameters like regularity index (RI) and base of support (BS) could be calculated. RI represents the ratio of the actual coordination to normal coordination of mice limbs. It was measured by the normal step sequence pattern (NSSP) and paw placement (PP) using the following formula: RI = (NSSP*4/PP) *100% [51]. During the movement, the more wrong gait sequences will lead to the lower RI value. Base of support, a sign of trunk stability, refers to the distance between the feet of the hind limbs perpendicular to the standing direction. The smaller the BS, the better the stability of the animal’s trunk.

### Electrophysiological assessment

Electrical activity was assessed at 4 weeks post injury as previously stated [52]. Briefly, the spinal cord was re-exposed on each group (*n* = 3 mice for each group). An electromyography machine (Medtronic, Minneapolis, MN, USA) was then used to evaluate their electrical activity. We put the bi-polar stimulating electrode into the spinal cord at the T6 spinal segment, and the recording electrode at the T13 spinal segment so as to record the action potentials including their peak amplitude and latency.

### Statistical analysis

The statistical analysis was performed using SPSS 19.0 (Chicago, IL, USA). Values were presented as mean ± standard deviation. Comparisons between different groups were conducted by Student’s *t*-test or one-way ANOVA followed by Bonferroni post-hoc test for multiple comparisons if data were in line with the normal distribution and the variances were homogeneous. If not, non-parametric tests would be performed. *P* < 0.05 was deemed to be statistically significant.

## Results

### Isolation, characterization, and label of 3D-HPMSCs

**Fig. 1:**
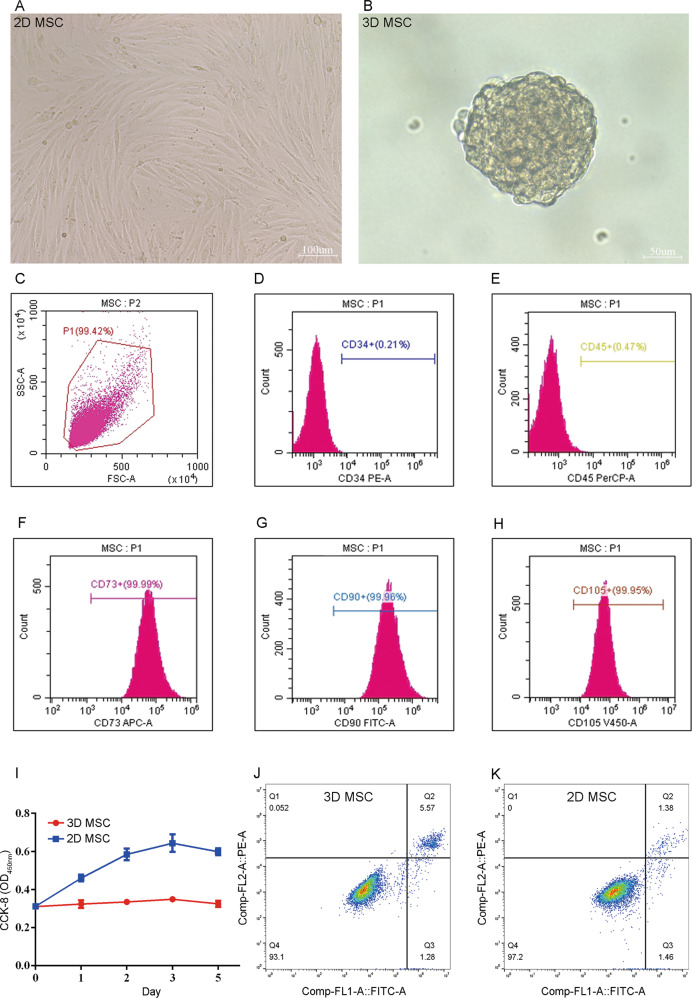
Isolation and characterization of 2D and 3D-HPMSCs. **A, B** The morphology of 2D and 3D-HPMSCs. Scale bar, 100 µm (**A**), and 50 µm (**B**)**. C** The flow-activated cytometric sorting. 99.42% MSCs were chosen for cell surface antigen analysis. **D–H** These selective cells expressed the CD73 (**F**), CD90 (**G**), and CD105 (**H**), but not CD34 (**D**), and CD45 (**E**). **I** Cells proliferation of 3D-HPMSCs was lower than that of 2D-HPMSCs, *P* < 0.05. **J, K** the apoptosis rate of 3D-HPMSCs and 2D-HPMSCs was 5.57% and 1.38%, respectively.

Primary HPMSCs were successfully isolated from the placenta and cultured in vitro. The monolayer-culture 2D-HPMSCs demonstrated a typical fibroblast-like, spindle-shaped morphology while the spheroid-culture 3D-HPMSCs aggregated into spheres and showed smaller cell size when they were digested into single cell (average diameter of 3D vs. 2D-HPMSCs: 10.08 ± 1.63 vs. 16.33 ± 2.66 μm, *P* < 0.0001) (Fig. 1A, B and Sup. Figure 1A–D). For better understanding the cell spheroidization, we recorded the dynamic process of spheroid formation in real time. Begun with scattered cells or small cell clumps, 3D-HPMSCs continuously recruited the cells around. After 2 h culture, 3D-HPMSCs started to display the preliminary spherical structure. About 16 h post 3D culture, HPMSCs finally aggregated into a standard sphere (Sup. Video 1 and 2).

The expression of cell surface antigens was further analyzed. As shown in Fig. 1C–H and Sup. Fig. 2A–F, cells expressed the markers of MSCS including CD73, CD90 and CD105, but not the hematopoietic or endothelial ones like CD34 and CD45, which complied with the ISCT definition criteria of MSC [53]. After confirming the isolated cells type, we managed to label the target cells with GFP fluorescence so as to track and analyze the transplanted cells in the next experiments (Sup. Fig. 2G, H).

### 3D-HPMSCs showed slightly lower cell proliferation and higher cell apoptosis

To further analyze the effects of 3D spheroid culture on cell viability, we conducted CCK8 test and flow cytometric analysis. CCK8 assay showed that the proliferation of HPMSCs were relatively inhibited after 3D culture (Fig. 1I), and flow cytometric analysis also indicated that cell apoptotic rate of 3D-HPMSCs were slightly higher than that of 2D-HPMSCs (Fig. 1J, K, 5.57% *vs* 1.38%). These results were somewhat expected, as spheroid culture would inevitably put a certain proportion of cells in the center of cell aggregation, and therefore kept them under a hypoxic or nutrient-deficient environment, resulting in slightly lower proliferation and higher apoptosis of 3D-HPMSCs.

### 3D-HPMSCs expression profile of the inflammatory factors, chemokine and trophic factors

**Fig. 2:**
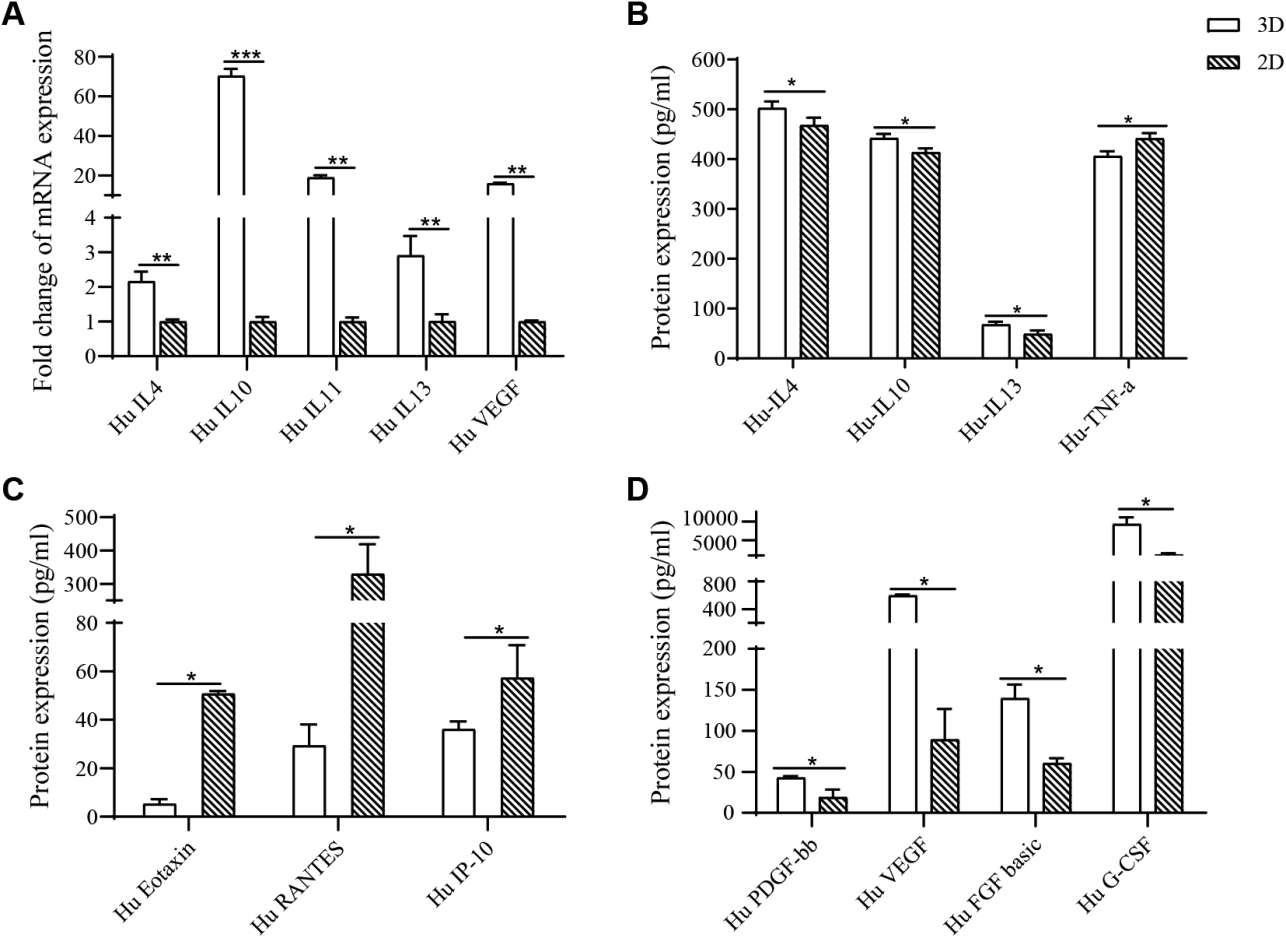
HPMSCs expression profile of cytokines. **A** The analysis of MSC-secreted cytokines in terms of inflammation (IL-4, IL-10, IL-11 and IL-13) and angiogenesis (VEGF) through RT-PCR. **B** 3D-HPMSCs promoted the secretion of anti-inflammatory factors (IL-4, IL-10, and IL-13), and inhibited pro-inflammatory factor TNF-a secretion compared with 2D-HPMSCs. **C** 3D-HPMSCs inhibited the secretion of chemokine (Eotaxin, Rantes, and IP-10) compared to 2D-HPMSCs. **D** 3D-HPMSCs also contributed to the secretion of trophic factors (PDGF-bb, VEGF, FGF basic, and G-CSF). **B** Enzyme-linked immunosorbent assay, **C**, **D** Bio-Plex ProTM cytokine assay, ∗, ∗∗, and ∗∗∗ indicate *P* < 0.05, *P* < 0.01, and *P* < 0.001, respectively.

In order to assess whether 3D-HPMSCs would alter the gene or protein expression profile upon cytokines in terms of inflammation, chemokine or trophic factors, which play important roles in tissue repair, qPCR, ELISA and cytokine plex assay were performed to detect mRNA expressions and protein secretion, respectively. As shown in Fig. 2A, 3D-HPMSCs significantly upregulated the expressions of representative anti-inflammatory cytokines (such as IL-4, IL-10, IL-11, and IL-13) and trophic factors (like VEGF). At the protein level, shown in Fig. 2B–D, 3D-HPMSCs effectively promoted the secretion of anti-inflammatory factors (IL-4, IL-10, and IL-13), trophic factors (PDGF-bb, VEGF, FGF basic, and G-CSF), and inhibited the secretion of pro-inflammatory factor (TNF-a), and chemokines (Eotaxin, RANRES, and IP-10). These results revealed that 3D spheroid culture could make HPMSCs more promising for anti-inflammation and tissue repair.

### 3D-HPMSCs contributed to angiogenesis in HUVECs, and attracted and promoted neurites growth in DRGs

**Fig. 3:**
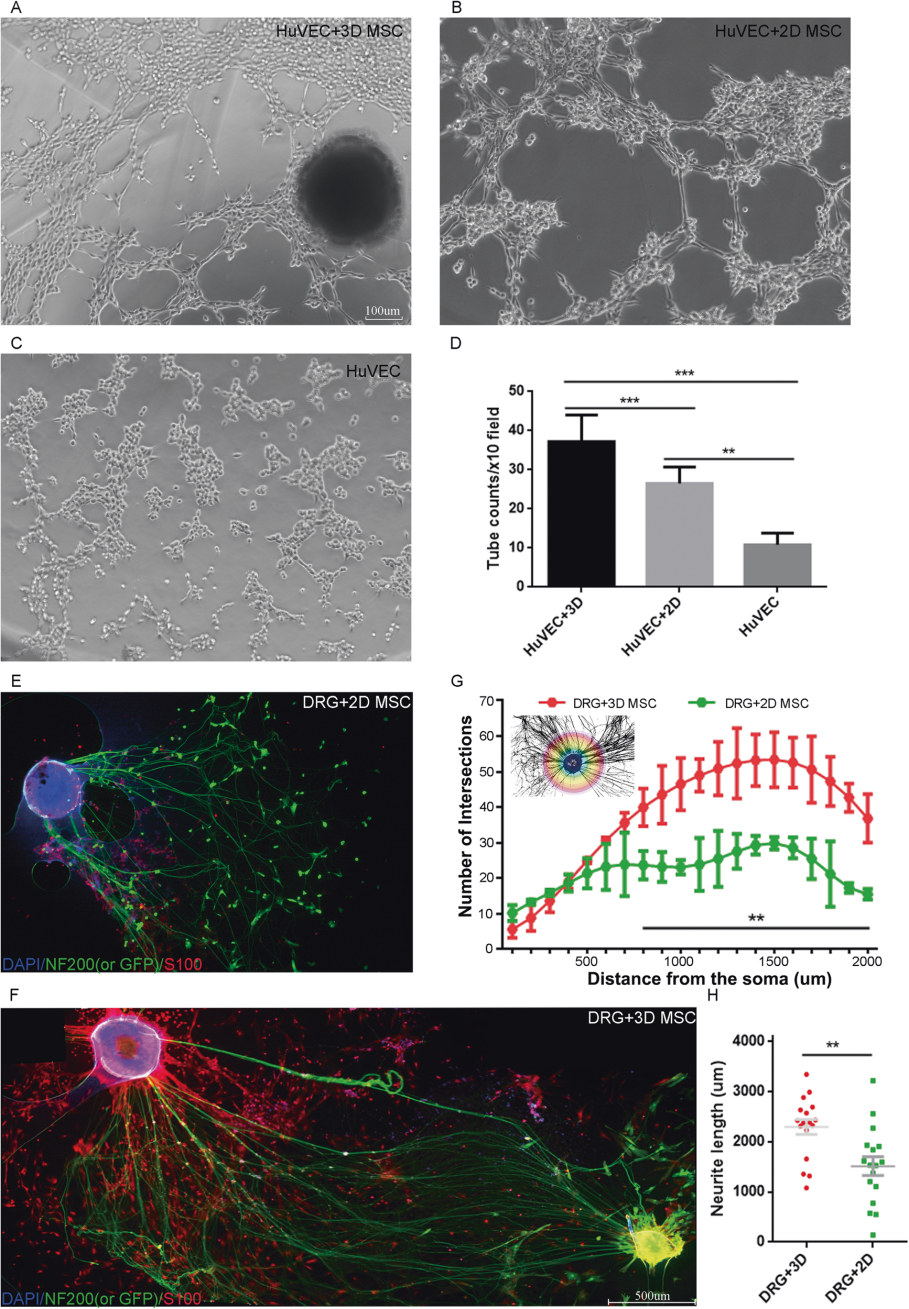
The effects of 3D-HPMSCs on angiogenesis and neurite morphogenesis. **A–C** Representative images showing the tube formation of HUVECs co-cultured with 3D-, 2D-HPMSCs, or medium alone. **D** There were more new vessel tubes formed in the 3D-HPMSCs co-culture group. **E**, **F** The DRG neurite outgrowth co-cultured with 2D and 3D-HPMSCs. Scale bar, 500 µm (E, F). **G** More branch points were observed in the 3D-HPMSCs co-culture as the distance increased from the soma. **H** Longer neurites were observed in the 3D-HPMSCs co-culture group. The number of branch points and the total length of neurites were collected using Sholl analysis (**G**) and simple neurite trace analysis (**H**). ∗∗, and ∗∗∗ indicate *P* < 0.01, and *P* < 0.001, respectively.

We next performed vessel tube formation assays and neurite growth Sholl analysis to test whether 3D-HPMSCs could promote angiogenesis and neurite morphogenesis. As shown in Fig. 3A–D, after incubation for 5.5 h, HUVECs co-cultured with 3D-HPMSCs generated more tube-like structure than those co-cultured with 2D-HPMSCs or medium alone. Supplementary Video 3 demonstrated the proangiogenic activities of 3D-HPMSCs. Then, to validate the effects of 3D-HPMSCs on neurite outgrowth, we managed to isolate dorsal root ganglion neurons(DRGs) from C57BL/6 newborn mice. After 7 days co-culture with HPMSCs, DRGs co-cultured with 3D-HPMSCs showed higher number of branch points and the total neurite distance when compared with DRGs co-cultured with 2D-HPMSCs (Fig. 3E–H, Sup. Figure 3A, B). Furthermore, DRGs had more neurite branches at the direction of 3D cell spheroid (Sup. Figure 3C–G).

### Transplanted cells survived and engrafted in the lesion area, and 3D-HPMSCs continued to secrete higher anti-inflammation and trophic factors

**Fig. 4:**
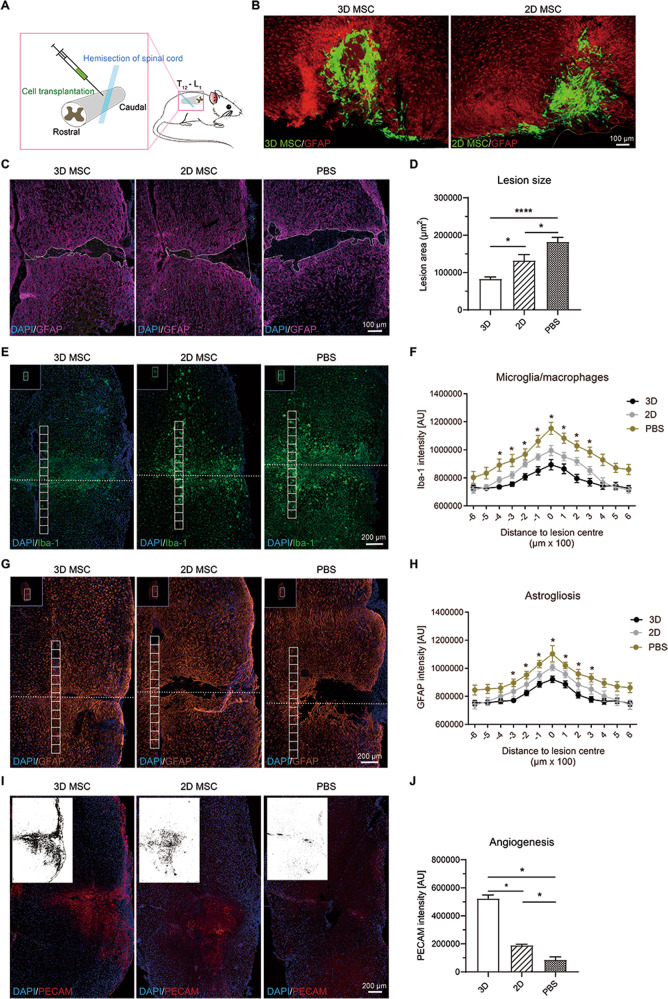
The neuroprotection of transplanted 3D-HPMSCs in injured spinal cord. **A** Schematic showing cell transplantation in a mouse model of spinal cord injury (SCI). **B** Representative images showing the survival of 3D- and 2D-HPMSCs in the spinal cord at 3 days after SCI. Scale bar, 100 µm. **C** Immunofluorescence showing lesion cavity of spinal cord in 3D-, 2D-HPMSCs and PBS groups at 4 weeks post SCI. Scale bar, 100 µm. **D** 3D-HPMSCs group showed significantly smaller lesion area at 4 weeks after SCI. **E**, Immunofluorescence showing inflammation in 3D-, 2D-HPMSCs and PBS groups at 4 weeks post SCI. Scale bar, 200 µm. **F** 3D-HPMSCs group showed significantly lower infiltration of microglia/macrophages at 4 weeks after SCI. **G** Immunofluorescence showing astrogliosis in 3D-, 2D-HPMSCs and PBS groups at 4 weeks post SCI. Scale bar, 200 µm. **H** 3D-HPMSCs group showed significantly lower astrogliosis at 4 weeks after SCI**. I** Immunofluorescence showing angiogenesis in 3D-, 2D-HPMSCs and PBS groups at 4 weeks post SCI. Scale bar, 200 µm. **J** 3D-HPMSCs group showed significantly better angiogenesis at 4 weeks after SCI. The upper left thumbnails in the (**E**), and (**G**) were the full view of the spinal cord. And the binary images in (**I**) stood for the density of blood vessels. ∗, and ∗∗∗ indicate *P* < 0.05, and *P* < 0.001, respectively.

Since 3D-HPMSCs had shown great potential on angiogenesis and neurite morphogenesis in vitro, we further investigated roles of 3D-HPMSCs in SCI mice in vivo (Fig. 4A). Three days after transplantation, GFP-positive cells could be observed in the lesion area of spinal cord (Fig. 4B), and continued to survive for the entire process of the experiment (Sup. Figure 4A–D). The majority of grafted cells tended to gather within the lesion epicenter while fewer were found to be interspersed in the perilesional area. However, it demonstrated a noteworthy decrease in cell survival in both groups at 7 days and 28 days post transplantation.

On the other hand, to determine whether transplanted 3D-HPMSCs could maintain their advantages in specific cytokines secretion, we performed ELISA on spinal cord at 28 days post injury. Similarly, as shown in Sup. Figures 4E, 3D-HPMSC-treated mice expressed higher level of anti-inflammation factors (IL-4, IL-10 and IL-13) and lower level of pro-inflammation factors (IL-6 and TNF-a) when compared with the 2D-HPMSC- and PBS-treated mice.

Thus, these results suggested that transplanted cells of both kinds managed to engraft in the spinal cord and survive at least 28 days, and 3D-HPMSCs were able to maintain their superiorities on secretion in vivo.

### Transplantation of 3D-HPMSCs exerted neuroprotection via decreasing the lesion cavity, inhibiting inflammation and astrogliosis, and promoting angiogenesis on the spinal cord

To determine the effects of 3D-HPMSCs on injured tissues protection, we then evaluated the size of lesion cavity, and the degree of astrogliosis and inflammation. GFAP, the biomarker of astrocytes in CNS system, could be used to distinguish the spared tissue against the lesion cavity [54, 55]. Therefore, we started by the measurement of lesion cavity size in each group at 4 weeks post injury using the GFAP staining. The cavity size of 3D-HPMSC-, 2D-HPMSC- and PBS-treated mice, which were outlined by the astrocytes margin (Fig. 4C), were 83029.15 ± 25635.88 um^2^, 132276.35 ± 55564.73 um^2^, and 182320.76 ± 50919.30 um^2^, respectively, indicating a significant reduction in the 3D-HPMSC-treated mice compared with 2D-HPMSC- and PBS-treated mice (Fig. 4D).

We then assessed the activation process of microglia/ macrophages and astrocytes after injury by the quantification of GFAP and Iba-1 intensity (see Methods in detail), which were typical hallmarks of neuropathology following SCI. As shown in Fig. 4E, F, 3D-HPMSC-treated mice exhibited a significant decrease in the presence of microglia/macrophages around the lesion core (from 400 um rostrally to 300 um caudally) compared with 2D-HPMSC- and PBS-treated controls. Similarly, as shown in Fig. 4G, H, 3D-HPMSC-treated mice showed a prominent reduction of GFAP intensity around the central zone of lesion (from 300 μm rostrally to 300 μm caudally).

We also analyzed the angiogenesis activities, which played an important role in tissue protection and repair through quantifying the PECAM intensity. At 28 days after injury, 3D-HPMSC-treated mice had more PECAM-labeled vessels around the lesion site than 2D-HPMSC- or PBS- treated ones (Fig. 4I, J)

Therefore, 3D-HPMSCs demonstrated great superiorities over 2D-HPMSCs and PBS on the neuroprotection in terms of the lesion cavity size, inflammation and astrogliosis response, and proangiogenic activities.

### Transplantation of 3D-HPMSCs ameliorated axons dieback, and improved functional outcomes after SCI

**Fig. 5:**
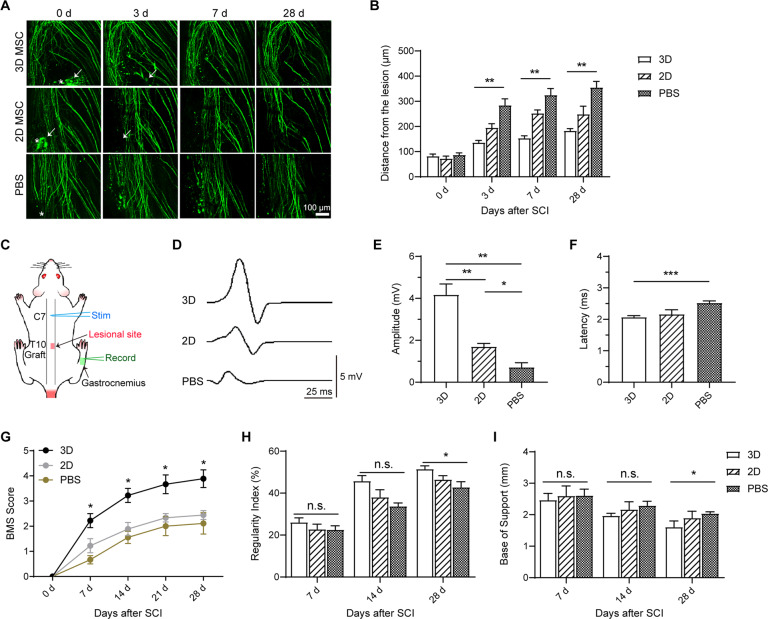
The therapeutic effects of grafted 3D-HPMSCs on axons and functional recovery. **A** The in vivo process of axonal dieback at 0 day, 3 days, 7 days, and 28 days post SCI via two-photon microscopy. Some transplanted cells were observed and indicated by the white arrows at 0 days and 3 days. Asterisk indicates the lesion site. Scale bar, 100 µm. **B** 3D-HPMSCs group showed significantly shorter axonal dieback after SCI. **C** The schematic illustration of electrophysiological evaluation. **D** Representative recording of action potentials in mice with 3D-, 2D-HPMSCs or PBS transplantation. **E** 3D-HPMSCs group showed higher amplitude compared with 2D-HPMSCs and PBS group at 28 days after SCI. **F** 3D-HPMSCs group showed shorter latency compared to PBS group at 28 days after SCI. **G** SCI mice with 3D-HPMSCs transplantation achieved higher BMS score compared to 2D-HPMSCs and PBS groups. **H** The regularity index in 3D-HPMSCs group was significantly higher than that in PBS group at 28 d post SCI. **I** The base of support test suggested a better functional improvement at 28 days post SCI. ∗, ∗∗, and ∗∗∗ indicate *P* < 0.05, *P* < 0.01, and *P* < 0.001, respectively.

Considering 3D-HPMSCs great advantages of neuroprotection, we further investigated whether these cells could rescue the damaged axons and then promoted functional recovery. We took advantage of two-photon microscope to longitudinally image the axonal dieback at 0, 3, 7, and 28 days post injury, which could provide us with the early dynamic changes of axons after SCI in vivo. As shown in Fig. 5A, axon-dieback distance quantification around the lesion site revealed a similar degree of axonal damage among three groups at the very beginning. Nevertheless, as time went on, 3D-HPMSC-, 2D-HPMSC- and PBS-treated mice demonstrated a distinct degree of axon dieback. The average axon dieback distances from the initial lesion site at 3, 7, and 28 days were 136.26 ± 11.98 μm, 153.77 ± 13.38 μm, and 183.04 ± 11.86 μm in 3D-HPMSC-treated mice, and 195.54 ± 34.62 μm, 252.83 ± 28.66 μm, and 249.28 ± 62.44 μm in 2D-HPMSC-treated mice, and 284.83 ± 56.01 μm, 325.13 ± 51.01, and 355.48 ± 47.32 μm in PBS-treated mice, respectively (Fig. 5B).

The functional recovery was then analyzed using electrophysiology, BMS locomotion test, and Catwalk step analysis. For electrophysiology evaluation, APs amplitude and latency were calculated and compared among three groups (Fig. 5C, D). As shown in Fig. 5E, F, animals treated with PBS presented very small response at 28d post injury, while mice treated with 3D- and 2D-HPMSCs showed much higher amplitude and lower latency of APs (3D-HPMSC-treated group were better than 2D-HPMSC-treated group), indicating both 3D- and 2D-HPMSCs have capacity to rescue partially functional connections after SCI.

For locomotion test, BMS score was calculated for four consecutive weeks post injury. As shown in Fig. 5G, all hind limbs on the injured side showed immediate paralysis after SCI, but followed by recovery to varying degrees in three groups. 3D-HPMSC-treated mice exhibited a progressive increase in the motor function of hind limb and demonstrated a significantly higher BMS scores at following time points in comparison with 2D-HPMSC- and PBS-treated mice.

For catwalk step analysis, RI (a measure of coordination) and base of support (a sign of trunk stability) were recorded and calculated. As shown in Fig. 5H, I, coordination and trunk stability of mice were severely affected after SCI, and showed mild, if any, spontaneous recovery at 14 day and 28 day time points in PBS-treated group. However, 3D-HPMSC-treated mice displayed a higher RI value and shorter length of base of support than 2D-HPMSC- and PBS-treated ones.

Taken together, these data implied that 3D-HPMSCs could alleviate the injured axon dieback and were conducive to functional recovery than 2D-HPMSCs and PBS.

### RNA-seq analysis of 3D-HPMSCs against 2D-HPMSCs

To figure out the potential mechanisms of 3D-HPMSCs better performances on cytokines secretion, and tissue protection and repair, we next performed RNA-sequencing on 3D and 2D-HPMSCs. Sup. Table 1 summarized the quality control results of RNA-Seq and proved data’s reliability.

**Fig. 6:**
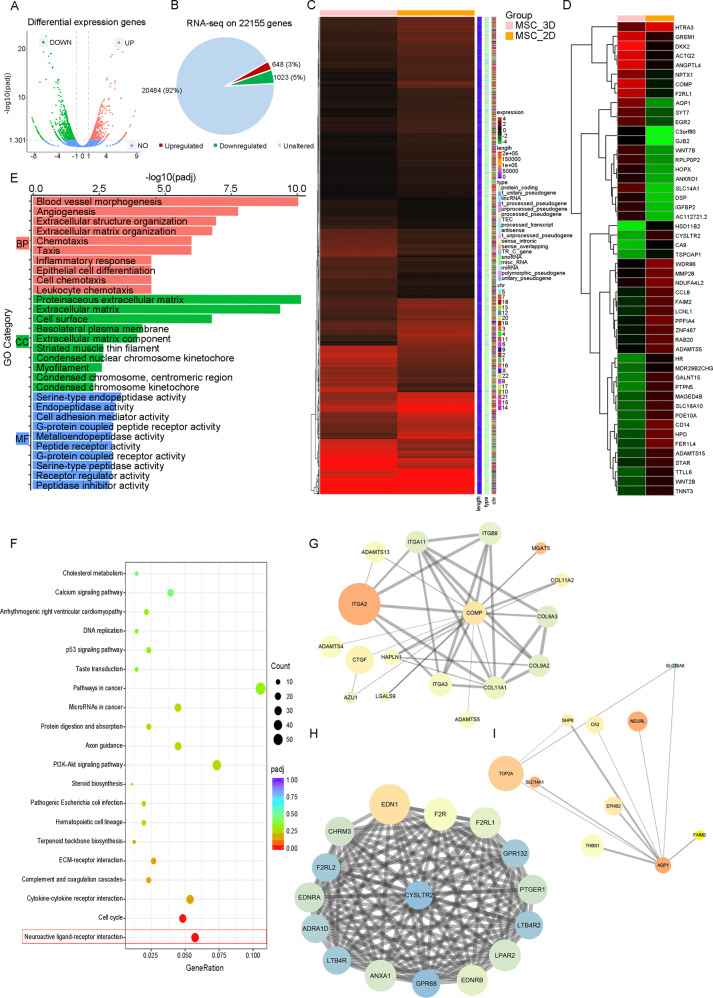
RNA-Seq analysis of 3D-HPMSCs-modulated genes. **A** The volcano plot illustrated the differentially expression genes between 3D- and 2D-HPMSCs. Red and green denote the upregulation and downregulation, respectively. **B**,Among 22155 genes, 648 genes were upregulated and 1023 genes were downregulated. **C**, **D** All of the significantly differentially expressed genes, and representative top 50 genes after the hierarchical clustering analysis. **E** The top 10 of most significantly affected categories in biological process (BP), cellular component (CC) and molecular function (MF) by Gene ontology functional clustering on differentially expressed genes. **F** The top 20 of most significantly affected pathway by KEGG pathway analysis of differentially expressed genes. **G–I** The protein-protein network analysis of representative two upregulated genes COMP, and AQP1, and one downregulated CYSCTR2.

Among the 1671 significantly differentially expressed genes (DEG), there were 648 upregulated genes and 1023 downregulated genes in 3D-HPMSCs compared with 2D-HPMSCs (Fig. 6A, B). All the DEGs were displayed in the hierarchical clustering heatmap (Fig. 6C), and the representative top 50 DEGs were shown in Fig. 6D and Sup. Table 2 and 3. Furthermore, GO enrichment analysis revealed that those significantly altered genes were mainly associated with blood vessel morphogenesis and angiogenesis (such as AQP1 and ANGPTL4), extracellular structure organization and extracellular matrix organization (such as COMP and ITGA2), and inflammatory response (such as CYSLTR2 and CD14) (Fig. 6E, Sup. Fig. 5A–C and Sup. table 4). KEGG pathway analysis of DEGs were mainly enriched in Neuroactive ligand-receptor interaction (Fig. 6F and Sup. table 5). We then chose the representative DEGs—AQP1, ANGPTL4, COMP, ITGA2, CYSLTR2 and CD14 for further PPI network. We found that many genes directly linked to these six genes were associated with blood vessels morphogenesis, angiogenesis, structure organization, and inflammation response (Fig. 6G–I), further implying that angiogenesis process, structure organization, and neuroactive ligand-receptor interaction were closely involved in 3D-HPMSCs structure formation and functional performance.

## Discussion

In this study, we reported the acquisition and characterization of 3D-HPMSCs as candidates for SCI repair. Previous studies have shown biomaterials-based 3D culture system was limited to good biocompatibility and degradation of the material [31, 56]. Here we showed a simple, easy-to-use, and high-efficiency strategy—3D-spheroid material-free culture system, enabling HPMSCs to aggregate into spheroids and retain neuroprotective and neurogenic capacity.

In the series of in vitro experiments, we found that 3D-HPMSCs showed a different cell morphology and behavior pattern: they aggregated up and formed spheroid-shape structures in the culture plate. When digested into single cells, they were of smaller cell size, which might be of particular significance as this characteristic would be conducive to significant decrease in lung entrapment after intravenous infusion of cells suspension [57, 58]. Moreover, 3D-HPMSCs exhibited excellent properties in secretion of anti-inflammatory and trophic factors, showing a remarkable autocrine and paracrine activity. These findings were further confirmed by the co-culture between 3D-HPMSCs with HUVECs or DRGs, in which 3D-HPMSCs contributed to more vessel tubes formation, and neurite outgrowth and branching.

When transplanted into the injured spinal cord, 3D-HPMSCs also demonstrated mighty effects on mice with SCI. They were able to survive for the entire experiment and engrafted in the lesion area, and maintain their advantageous properties in high secretion of anti-inflammation and trophic factors. Histopathological analysis indicated that 3D-HPMSC-treated mice exerted prominent neuroprotection on the spinal cord by minimizing the lesion cavity, inhibiting inflammation and astrogliosis, and promoting angiogenesis on the spinal cord when compared with 2D-HPMSC- and PBS-treated mice.

Previous studies indicated that inflammation and astrogliosis took important parts in pathological process after SCI [47, 59–62,]. For one thing, overactive inflammatory reaction at the lesion site could directly aggravate the secondary damage of spinal cord and then lead to an exacerbated prognosis of SCI. For another, reactive astrogliosis, characterized by the increase in GFAP, intermediate filaments, and vimentin, would develop following the activation of microglia/macrophages after injury [63], which built a growth-inhibiting milieu and, therefore, affected tissue repair. Overactive astrocytes also produced varieties of inhibitory molecules like chondroitin sulfate proteoglycans [64], producing a physical barrier impairing axonal regeneration. As a result, the capacity to upregulate anti-inflammatory factors and downregulate pro-inflammatory factors enabled 3D-HPMSCs to regulate the overactive inflammation and reactive astrogliosis after SCI. And combined with high expression of trophic factors like PDGF, VEGF and FGF (beneficial for spared neuronal protection [60, 61], axonal regeneration [62, 65], angiogenesis [66], etc.), 3D-HPMSCs would then create a positive environment beneficial for tissue repair and hopefully improve the functional recovery post SCI.

To further support the findings above, we took advantage of two-photon microscope to evaluate the dynamic changes of axons over multiple days. In general, microglia or infiltrated macrophages would migrate to the axons and greatly induce axonal retraction, which was known as axon dieback [67]. This exacerbated the secondary injury and increased functional deficits. Our data showed that although axon dieback generally existed among three groups, 3D-HPMSC-treated mice showed a significant decrease in axonal dieback distance compared with 2D-HPMSC- or PBS-treated mice, indicating that treatment with 3D-HPMSCs might be conducive to attenuating progressive axon dieback and protecting the spared axons post injury. Further electrophysiological evaluation confirmed the better electrical activities of spared axons in 3D-HPMSC-treat mice. BMS score and Catwalk step pattern analysis also showed better functional recovery and good body coordination and trunk stability in 3D-HPMSCs group.

Finally, we investigated the molecular mechanisms responsible for the significant difference between 3D- and 2D-HPMSC using the RNA-Seq analysis. 3D-HPMSCs significantly altered the expression of genes associated with blood vessel morphogenesis, angiogenesis, extracellular structure organization, and extracellular matrix organization, inflammatory response, and were closely involved in the pathway of Neuroactive ligand-receptor interaction. Specifically, 3D-HPMSCs significantly upregulated the genes associated with angiogenesis [68, 69] and structure organization [70, 71], and downregulated the genes related to pro-inflammatory response [72, 73], which were consistent with our findings above. Moreover, we speculated that significant changes in genes expression related to angiogenesis and inflammation regulation might be attributed to the extracellular structure/matrix organization, since 3D-spheroid culture forced HPMSCs to aggregate into spheroids and remodel cells structure in the beginning. This speculation was based on the previous studies [74, 75], which stated that 3D culture increased pluripotent genes expression mainly because of the structure reorganization--relaxation of cytoskeleton tension.

It should be noted that there are some limitations in our study. Firstly, 3D spheroid culture seemed to inevitably affect cells viability to a slight extent. These were anticipated since the living space of spheroid structure was limited and cells in the core site might be short of nutrition as well as oxygen as time went on. Secondly, though survived for the duration of experiment, transplanted cells showed a gradual reduction of cell density within the injured site. This compromised survival of grafted cells left us a question of whether the well-known immunosuppressive properties of MSCs [76] was sufficient to prevent xenograft rejection. Maybe immunosuppression drugs were needed for HPMSCs transplantation into mice. Lastly, we conducted a preliminary study on MSCs differentiation into neuron-like cells, and found no Tuji^+^ cells after Tuji immunofluorescence staining. But as we had not made an in-depth study on that, we would better draw no conclusion on HPMSCs ability to trans-differentiate into neuron-like cells.

## Conclusion

To our knowledge, our experiment might be the first attempt to evaluate the therapeutic effects and underlying mechanisms of 3D-spheroid cultured HPMSCs on the spinal cord repair in vitro and in vivo. We found that 3D-HPMSCs group demonstrated excellent properties in secretion of anti-inflammatory and trophic factors, and great potentials on angiogenesis and neurite morphogenesis in vitro. After transplantation into the injured spinal cord, 3D-HPMSCs managed to survive for the entire experiment and maintain their advantageous properties in secretion. Further, 3D-HPMSCs exhibited remarkable effects on neuroprotection and mice treated with 3D-HPMSCs obtained substantial improvement of functional recovery. RNA-Seq suggested that extracellular structure/matrix organization of 3D-HPMSCs might increase pluripotent genes expression on angiogenesis, and inflammation regulation. These promising results indicate that 3D-HPMSCs hold great potentials for cell-based therapy on SCI.

## Supplementary information

Sup Fig legends

Sup Fig 1

Sup Fig 2

Sup Fig 3

Sup Fig 4

Sup Fig 5

Sup. table 1

Sup. table 2

Sup. table 3

Sup. table 4

Sup. table 5

Video 1

Video 2

Video 3

Checklist

Author Contribution Statement

## Data Availability

Datasets analyzed during the current study are available from the corresponding author on reasonable request. The RNA-Seq data have been deposited and released at Gene Expression Omnibus (GEO) (http://www.ncbi.nlm.nih.gov/geo/) under accession ID GSE174619.

## Code availability

The code to reproduce GO and KEGG enrichment analysis in the paper is available and a brief user guide can be requested from the authors.
