# Supplementary material for: 3D spheroids of human placenta-derived mesenchymal stem cells attenuate spinal cord injury in mice
Source: Cell Death Dis. 2021 Nov 22;12(12):1096. doi: 10.1038/s41419-021-04398-w (PMC8606575; doi:10.1038/s41419-021-04398-w)
Supplement: Supplementary file 1 — Sup Fig legends [file 41419_2021_4398_MOESM1_ESM.docx]

**Sup Fig 1 Cell size of the 3D and 2D HPMSCs in vitro. A, B,** The morphology of 2D and 3D HPMSCs after trypsinization in brightfield. Scale bars=100 μm. **C, D,** The average diameter and size distribution of 2D and 3D HPMSCs. The data were expressed as mean ± SD. ∗∗∗ indicates *P* < 0.001.

**Sup Fig 2 Cell phenotype of the isotype control of MSCs and the labeling of MSCs using GFP-lentivirus. A-F,** The isotype control did not express MSC marker. **G, H,** The MSCs morphology with or without fluorescence. Scale bar=200um (**G**), and scale bar=100um (**H**).

**Sup Fig 3 3D HPMSCs attracted neurite outgrowth and branching. A, B,** The simple neurite trace analysis of neurite length of DRGs co-cultured with 3D HPMSCs or 2D HPMSCs. About 15-20 neurites would be chosen for calculation in each DRG. **C, D,** The Sholl analysis process of branch points of DRGs co-cultured with 3D HPMSCs or 2D HPMSCs. **E,** **F,** At the direction of HPMSCs, DRGs co-cultured with 3D HPMSCs showed more branches when compared with that co-cultured with 2D HPMSCs. The color atlas represented the number of branch points. **G,** The Sholl analysis indicated that more branch points of DRG appeared in 3D HPMSCs co-culture group.

**Sup Fig 4 3D HPMSCs survived for the entire experiment and maintained their anti-inflammatory properties in vivo.** **A and B, and C and D,** Cells survival of the 2D and 3D HPMSCs at 7d and 28d post injury, respectively. **E,** The representative expression profile of inflammation (IL-4, IL-10, IL-13, IL-6 and TNF-a) in the spinal cord among three groups using ELISA. ∗ indicates *P* < 0.05, ∗∗ indicates *P* < 0.01, and ∗∗∗ indicates *P* < 0.001. Scale bar=100um (**A-D**).

**Sup Fig 5 Protein-protein network analysis** **of representative genes.** **A, B,** The upregulated gene ITGA2 (related to structure organization), and ANGPTL4 (related to angiogenesis). **C,** The downregulated gene CD14 (related to pro-inflammatory response).

**Sup Video 1 and Video 2** represented the dynamic process of 3D HPMSCs formation under brightfield and immunofluorescence microscope.

**Sup Video 3** showed the tube formation process of HUVECs co-cultured with 3D HPMSCs.
