# Supplementary figures and images for: 3D spheroids of human placenta-derived mesenchymal stem cells attenuate spinal cord injury in mice
Source: Cell Death Dis. 2021 Nov 22;12(12):1096. doi: 10.1038/s41419-021-04398-w (PMC8606575; doi:10.1038/s41419-021-04398-w)

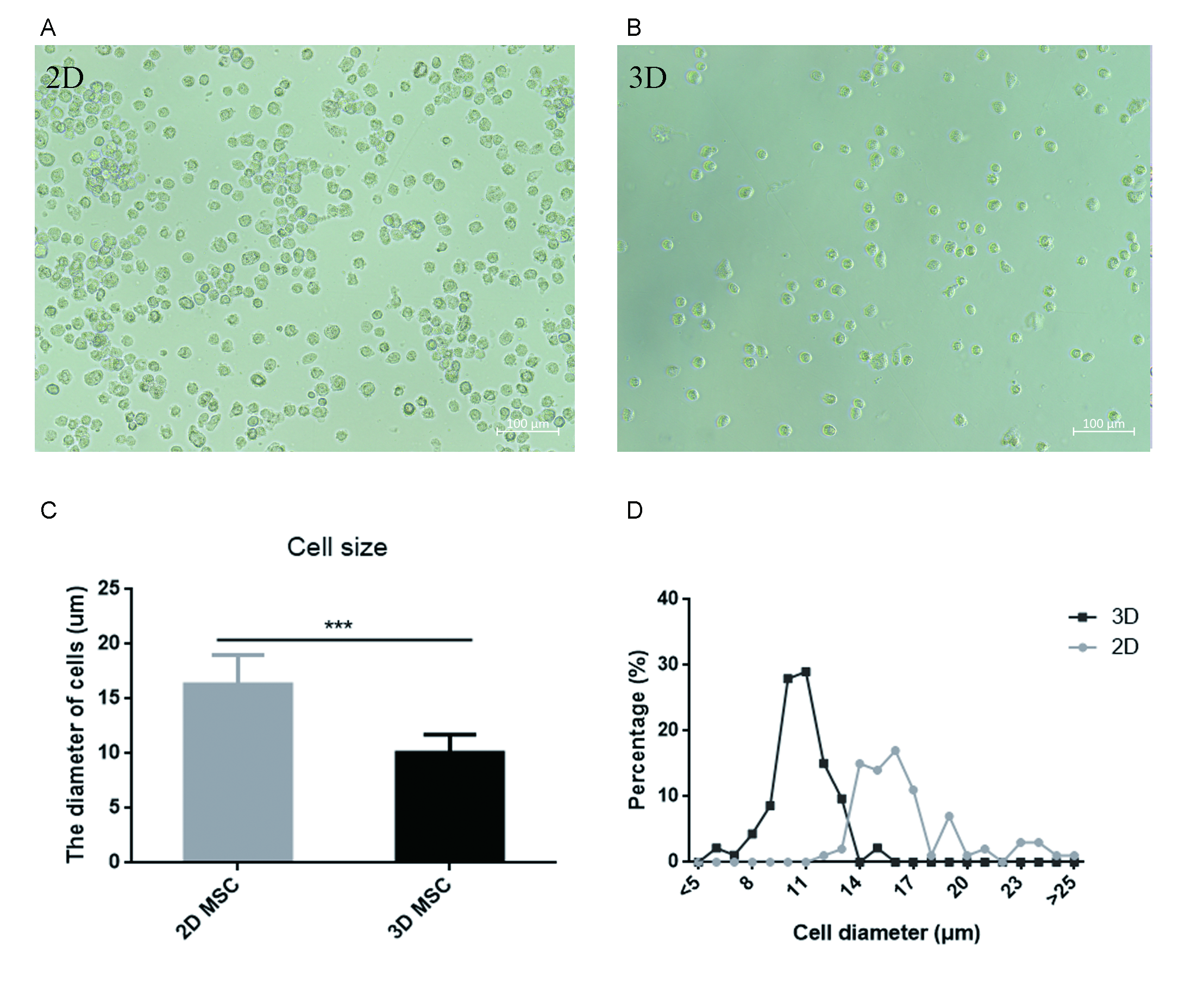

Supplement: Supplementary file 2 — Sup Fig 1 [file 41419_2021_4398_MOESM2_ESM.tif]

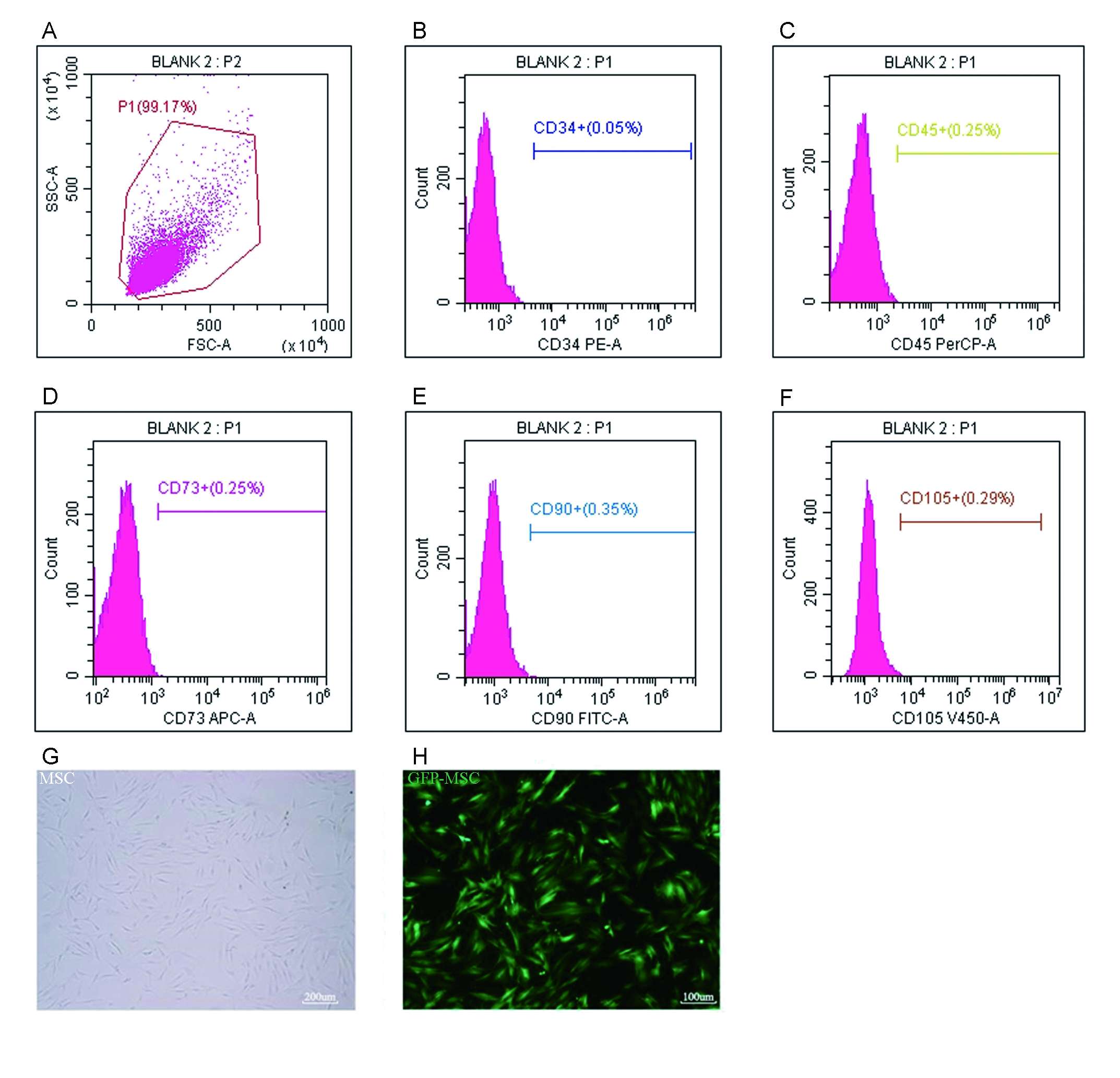

Supplement: Supplementary file 3 — Sup Fig 2 [file 41419_2021_4398_MOESM3_ESM.tif]

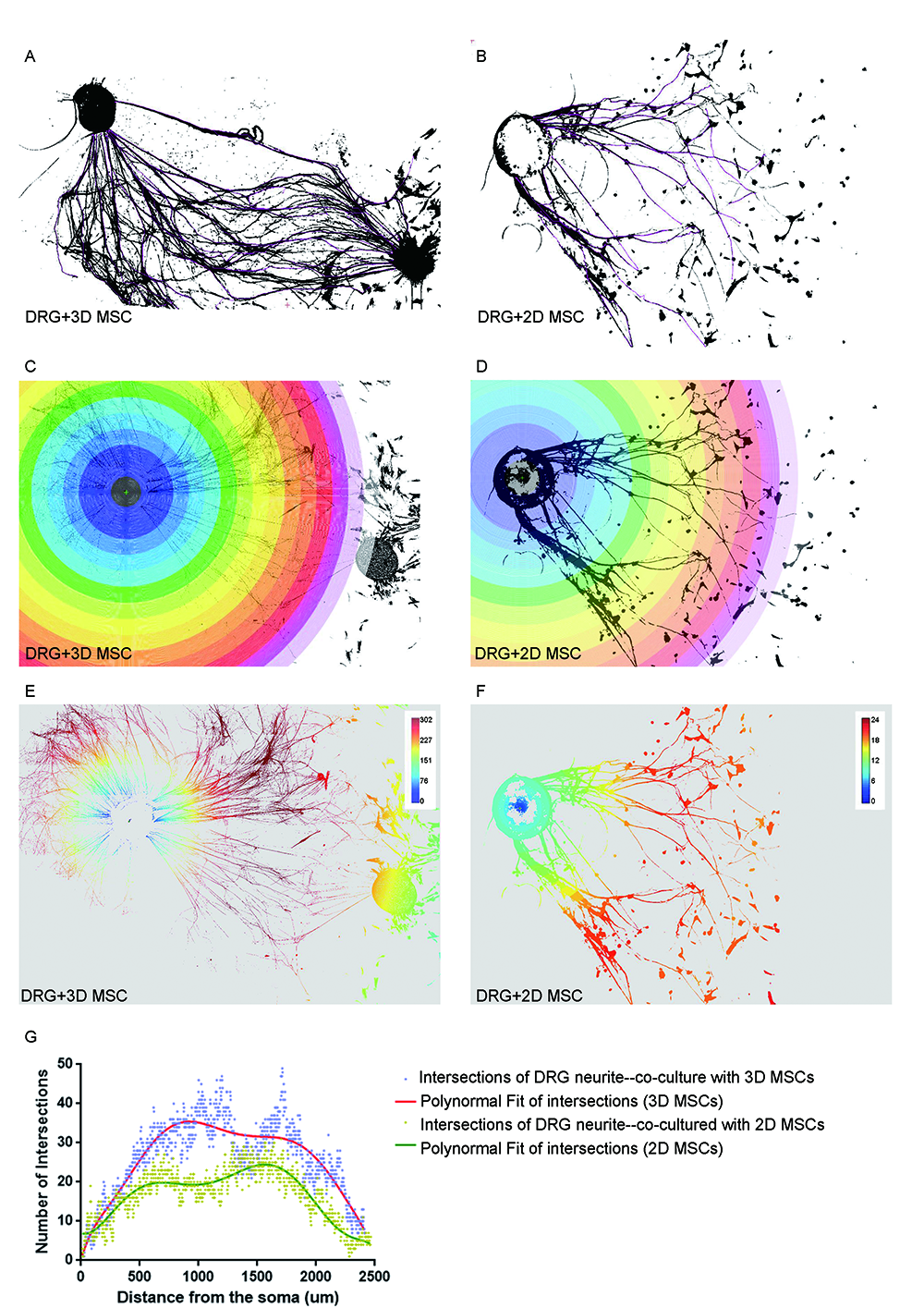

Supplement: Supplementary file 4 — Sup Fig 3 [file 41419_2021_4398_MOESM4_ESM.tif]

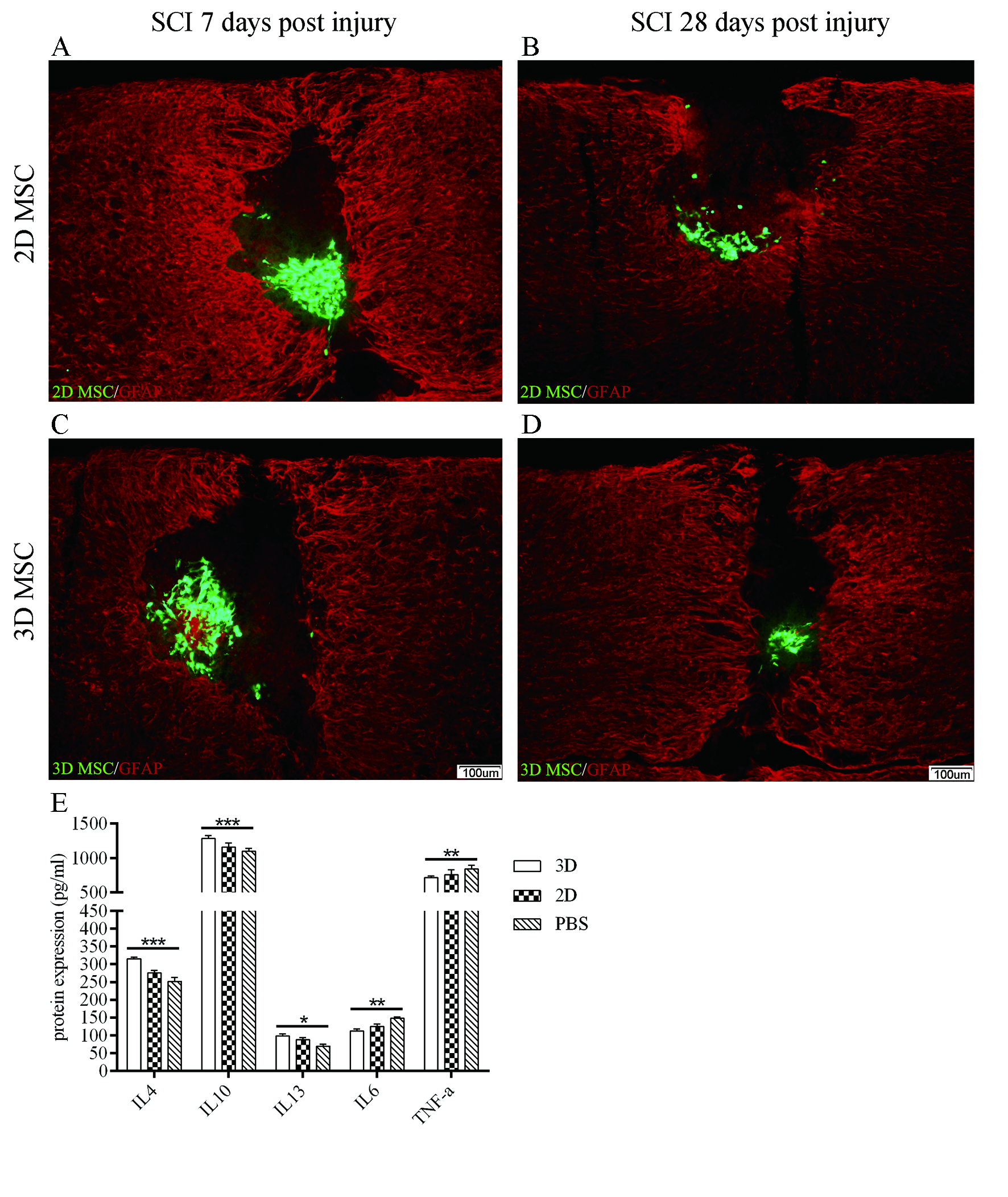

Supplement: Supplementary file 5 — Sup Fig 4 [file 41419_2021_4398_MOESM5_ESM.tif]
