# Supplementary material for: 3D spheroids of human placenta-derived mesenchymal stem cells attenuate spinal cord injury in mice
Source: Cell Death Dis. 2021 Nov 22;12(12):1096. doi: 10.1038/s41419-021-04398-w (PMC8606575; doi:10.1038/s41419-021-04398-w)
Supplement: Supplementary file 7 — Sup. table 1 [file 41419_2021_4398_MOESM7_ESM.docx]

Sup. table 1 Quality control summary of the RNA sequence results of MSC samples

| Group | Total_reads | Total_map | Unique_map | Multi_map | Q30 | GC_pct |
| --- | --- | --- | --- | --- | --- | --- |
| MSC_2D | 44074124 | 42682571(96.84%) | 41238150(93.57%) | 1444421(3.28%) | 95.24% | 53.98% |
| MSC_3D | 43032244 | 41934614(97.45%) | 40731416(94.65%) | 1203198(2.8%) | 95.44% | 53.17% |

Total reads: The number of clean reads after filtering the original data

Total map: The number and percentage of reads to the reference genome.

Uniq map: The number and percentage of reads to the unique location of the reference genome.

Multiple map: The number and percentage of reads to multiple locations in the reference genome.

Q30: The percentage of bases with a Clean Data mass value ≥ 30.

GC pct: The percentage of G and C bases in Clean Data to the total bases.
