# Supplementary material for: 3D spheroids of human placenta-derived mesenchymal stem cells attenuate spinal cord injury in mice
Source: Cell Death Dis. 2021 Nov 22;12(12):1096. doi: 10.1038/s41419-021-04398-w (PMC8606575; doi:10.1038/s41419-021-04398-w)
Supplement: Supplementary file 8 — Sup. table 2 [file 41419_2021_4398_MOESM8_ESM.docx]

Sup. table 2 List of the top 50 upregulated genes following 3D-spheroid culture

| Gene symbol | Gene description | Log2Foldchange | P value adjust |
| --- | --- | --- | --- |
| AQP1 | aquaporin 1 (Colton blood group) | 5.898141 | 1.15E-20 |
| SLC14A1 | solute carrier family 14 member 1 (Kidd blood group) | 6.673624 | 3.77E-20 |
| COMP | cartilage oligomeric matrix protein | 4.897258 | 1.46E-19 |
| DKK2 | dickkopf WNT signaling pathway inhibitor 2 | 4.734388 | 4.02E-19 |
| F2RL1 | F2R like trypsin receptor 1 | 4.148518 | 8.42E-15 |
| ACTG2 | actin, gamma 2, smooth muscle, enteric | 4.015591 | 1.77E-14 |
| ANGPTL4 | angiopoietin like 4 | 3.993592 | 2.11E-14 |
| IGFBP2 | insulin like growth factor binding protein 2 | 5.43982 | 3.36E-14 |
| AC112721.2 | novel protein (LOC728009) | 5.350866 | 9.30E-14 |
| WNT7B | Wnt family member 7B | 4.726713 | 1.19E-13 |
| RPLP0P2 | ribosomal protein lateral stalk subunit P0 pseudogene 2 | 4.564796 | 4.97E-13 |
| DSP | desmoplakin | 5.334312 | 1.53E-12 |
| C3orf80 | chromosome 3 open reading frame 80 | 6.419578 | 1.07E-10 |
| GJB2 | gap junction protein beta 2 | 6.405241 | 1.24E-10 |
| HOPX | HOP homeobox | 4.336859 | 1.24E-10 |
| SYT7 | synaptotagmin 7 | 3.604977 | 1.81E-10 |
| ANKRD1 | ankyrin repeat domain 1 | 4.363995 | 2.35E-10 |
| EGR2 | early growth response 2 | 3.535388 | 3.78E-10 |
| NPTX1 | neuronal pentraxin 1 | 3.280786 | 7.84E-10 |
| GREM1 | gremlin 1, DAN family BMP antagonist | 3.106838 | 2.10E-09 |
| TSLP | thymic stromal lymphopoietin | 3.337715 | 3.50E-09 |
| CA2 | carbonic anhydrase 2 | 4.335149 | 4.06E-09 |
| LINC01614 | long intergenic non-protein coding RNA 1614 | 4.477097 | 4.47E-09 |
| HHIP | hedgehog interacting protein | 3.2232 | 4.47E-09 |
| RCAN2 | regulator of calcineurin 2 | 3.580626 | 5.22E-09 |
| NTM | neurotrimin | 3.084476 | 5.65E-09 |
| LZTS1 | leucine zipper tumor suppressor 1 | 3.263819 | 5.94E-09 |
| ADAMTS14 | ADAM metallopeptidase with thrombospondin type 1 motif 14 | 3.023173 | 6.30E-09 |
| AC112721.1 | novel transcript | 4.064677 | 6.30E-09 |
| MMP11 | matrix metallopeptidase 11 | 3.014468 | 1.16E-08 |
| KCNS3 | potassium voltage-gated channel modifier subfamily S member 3 | 3.526117 | 1.20E-08 |
| USP44 | ubiquitin specific peptidase 44 | 3.773076 | 1.65E-08 |
| TAGLN | transgelin | 2.868894 | 2.45E-08 |
| HBEGF | heparin binding EGF like growth factor | 4.468932 | 3.50E-08 |
| RASSF2 | Ras association domain family member 2 | 4.013463 | 3.57E-08 |
| F2R | coagulation factor II thrombin receptor | 2.93401 | 4.40E-08 |
| E2F7 | E2F transcription factor 7 | 3.343243 | 4.44E-08 |
| AKR1B10 | aldo-keto reductase family 1 member B10 | 8.773791 | 4.72E-08 |
| RGS4 | regulator of G protein signaling 4 | 2.978935 | 5.75E-08 |
| ADAMTS16 | ADAM metallopeptidase with thrombos-pondin type 1 motif 16 | 4.625077 | 1.07E-07 |
| ITGA2 | integrin subunit alpha 2 | 2.755584 | 1.17E-07 |
| CNIH3 | cornichon family AMPA receptor auxiliary protein 3 | 3.280391 | 1.33E-07 |
| CNN1 | calponin 1 | 3.044009 | 1.38E-07 |
| HECW2 | HECT, C2 and WW domain containing E3 ubiquitin protein ligase 2 | 3.775458 | 1.44E-07 |
| EXTL1 | exostosin like glycosyltransferase 1 | 4.951245 | 2.06E-07 |
| TNFRSF12A | TNF receptor superfamily member 12A | 2.695855 | 2.26E-07 |
| TIMP3 | TIMP metallopeptidase inhibitor 3 | 2.651088 | 2.65E-07 |
| NES | nestin | 2.718876 | 3.02E-07 |
| SYTL5 | synaptotagmin like 5 | 8.490715 | 5.79E-07 |
| SPCS2P4 | signal peptidase complex subunit 2 pseudo-gene 4 | 3.264231 | 6.65E-07 |
