# Supplementary material for: 3D spheroids of human placenta-derived mesenchymal stem cells attenuate spinal cord injury in mice
Source: Cell Death Dis. 2021 Nov 22;12(12):1096. doi: 10.1038/s41419-021-04398-w (PMC8606575; doi:10.1038/s41419-021-04398-w)
Supplement: Supplementary file 9 — Sup. table 3 [file 41419_2021_4398_MOESM9_ESM.docx]

Sup. table 3 List of the top 50 downregulated genes following 3D-spheroid culture

| Gene symbol | Gene description | Log2Foldchange | P value adjust |
| --- | --- | --- | --- |
| CD14 | CD14 molecule | -6.09802 | 2.30E-23 |
| CYSLTR2 | cysteinyl leukotriene receptor 2 | -6.52398 | 3.88E-20 |
| HPD | 4-hydroxyphenylpyruvate dioxygenase | -5.32017 | 7.45E-20 |
| FER1L4 | fer-1 like family member 4, pseudogene | -5.24784 | 8.80E-20 |
| GALNT15 | polypeptide N-acetylgalacto-saminyl-transferase 15 | -5.67682 | 2.74E-19 |
| PTPN5 | protein tyrosine phosphatase, non-receptor type 5 | -5.51179 | 7.12E-18 |
| SLC16A10 | solute carrier family 16 member 10 | -5.01458 | 4.50E-17 |
| PDE10A | phosphodiesterase 10A | -4.97724 | 7.23E-17 |
| CA9 | carbonic anhydrase 9 | -6.06685 | 1.55E-14 |
| HTRA3 | HtrA serine peptidase 3 | -3.98083 | 1.65E-14 |
| PPFIA4 | PTPRF interacting protein alpha 4 | -4.1332 | 2.17E-14 |
| WDR86 | WD repeat domain 86 | -4.01804 | 4.14E-14 |
| HSD11B2 | hydroxysteroid 11-beta dehydrogenase 2 | -7.10149 | 5.82E-14 |
| MAGED4B | MAGE family member D4B | -4.42744 | 5.82E-14 |
| FAIM2 | Fas apoptotic inhibitory molecule 2 | -4.12273 | 1.05E-13 |
| ADAMTS15 | ADAM metallopeptidase with thrombospondin type 1 motif 15 | -4.10924 | 7.60E-13 |
| HR | HR, lysine demethylase and nuclear receptor corepressor | -4.72376 | 8.29E-13 |
| LCNL1 | lipocalin like 1 | -3.87079 | 1.84E-12 |
| RAB20 | RAB20, member RAS oncogene family | -3.71312 | 6.88E-12 |
| TTLL6 | tubulin tyrosine ligase like 6 | -3.96935 | 3.01E-11 |
| CCL8 | C-C motif chemokine ligand 8 | -3.60158 | 4.97E-11 |
| MMP28 | matrix metallopeptidase 28 | -3.34952 | 3.13E-10 |
| ADAMTS5 | ADAM metallopeptidase with thrombospondin type 1 motif 5 | -3.39963 | 3.13E-10 |
| NDUFA4L2 | NDUFA4, mitochondrial complex associated like 2 | -3.31923 | 3.13E-10 |
| MIR29B2CHG | MIR29B2 and MIR29C host gene | -3.90315 | 3.78E-10 |
| ZNF467 | zinc finger protein 467 | -3.36607 | 5.93E-10 |
| STAR | steroidogenic acute regulatory protein | -3.49199 | 6.03E-10 |
| WNT2B | Wnt family member 2B | -3.61443 | 7.67E-10 |
| TNNT3 | troponin T3, fast skeletal type | -3.48053 | 2.09E-09 |
| TSPOAP1 | TSPO associated protein 1 | -4.4379 | 3.25E-09 |
| ITGB8 | integrin subunit beta 8 | -3.17142 | 3.40E-09 |
| NPR3 | natriuretic peptide receptor 3 | -3.07776 | 5.21E-09 |
| BST2 | bone marrow stromal cell antigen 2 | -3.05623 | 5.50E-09 |
| GAB2 | GRB2 associated binding protein 2 | -3.05953 | 5.98E-09 |
| ABCC6 | ATP binding cassette subfamily C member 6 | -3.59581 | 7.13E-09 |
| FZD4 | frizzled class receptor 4 | -2.99174 | 1.30E-08 |
| PPL | periplakin | -3.75165 | 2.76E-08 |
| HAPLN1 | hyaluronan and proteoglycan link protein 1 | -3.05422 | 2.98E-08 |
| NEAT1 | nuclear paraspeckle assembly transcript 1 | -2.81855 | 4.32E-08 |
| G0S2 | G0/G1 switch 2 | -2.8955 | 4.44E-08 |
| AC073335.2 | novel transcript | -3.30072 | 4.44E-08 |
| ADAMTS6 | ADAM metallopeptidase with thrombo-spondin type 1 motif 6 | -2.85377 | 4.71E-08 |
| ADAMTS1 | ADAM metallopeptidase with thrombo-spondin type 1 motif 1 | -2.78092 | 6.55E-08 |
| PTPRQ | protein tyrosine phosphatase, receptor type Q | -3.65552 | 7.27E-08 |
| CDON | cell adhesion associated, oncogene regulated | -2.95594 | 1.07E-07 |
| CCDC69 | coiled-coil domain containing 69 | -2.79811 | 1.07E-07 |
| MEG9 | maternally expressed 9 | -2.82755 | 1.11E-07 |
| SSPO | SCO-spondin | -3.34674 | 1.17E-07 |
| CLMN | calmin | -3.39518 | 1.17E-07 |
| XPNPEP2 | X-prolyl aminopeptidase 2 | -5.07508 | 1.17E-07 |
