# Supplementary material for: 3D spheroids of human placenta-derived mesenchymal stem cells attenuate spinal cord injury in mice
Source: Cell Death Dis. 2021 Nov 22;12(12):1096. doi: 10.1038/s41419-021-04398-w (PMC8606575; doi:10.1038/s41419-021-04398-w)
Supplement: Supplementary file 10 — Sup. table 4 [file 41419_2021_4398_MOESM10_ESM.docx]

Sup. table 4 List of the top 50 GO terms following 3D-spheroid culture

| Go ID | Go description | Go category | P value adjust |
| --- | --- | --- | --- |
| GO:0005578 | proteinaceous extracellular matrix | CC | 7.06E-11 |
| GO:0048514 | blood vessel morphogenesis | BP | 9.26E-11 |
| GO:0031012 | extracellular matrix | CC | 4.38E-10 |
| GO:0001525 | angiogenesis | BP | 1.74E-08 |
| GO:0043062 | extracellular structure organization | BP | 1.19E-07 |
| GO:0030198 | extracellular matrix organization | BP | 1.65E-07 |
| GO:0009986 | cell surface | CC | 1.69E-07 |
| GO:0006935 | chemotaxis | BP | 1.01E-06 |
| GO:0042330 | taxis | BP | 1.01E-06 |
| GO:0030855 | epithelial cell differentiation | BP | 3.32E-05 |
| GO:0006954 | inflammatory response | BP | 3.32E-05 |
| GO:0060326 | cell chemotaxis | BP | 3.32E-05 |
| GO:0030595 | leukocyte chemotaxis | BP | 3.32E-05 |
| GO:0050920 | regulation of chemotaxis | BP | 3.70E-05 |
| GO:0016323 | basolateral plasma membrane | CC | 7.18E-05 |
| GO:0044420 | extracellular matrix component | CC | 0.000112 |
| GO:0043410 | positive regulation of MAPK cascade | BP | 0.000115 |
| GO:0014706 | striated muscle tissue development | BP | 0.000116 |
| GO:0007517 | muscle organ development | BP | 0.000118 |
| GO:0070372 | regulation of ERK1 and ERK2 cascade | BP | 0.00015 |
| GO:0060537 | muscle tissue development | BP | 0.000172 |
| GO:0097529 | myeloid leukocyte migration | BP | 0.000172 |
| GO:1901342 | regulation of vasculature development | BP | 0.000172 |
| GO:0051146 | striated muscle cell differentiation | BP | 0.000252 |
| GO:0001501 | skeletal system development | BP | 0.000366 |
| GO:0061041 | regulation of wound healing | BP | 0.000409 |
| GO:0004252 | serine-type endopeptidase activity | MF | 0.000449 |
| GO:0045765 | regulation of angiogenesis | BP | 0.000577 |
| GO:0042692 | muscle cell differentiation | BP | 0.00061 |
| GO:0070371 | ERK1 and ERK2 cascade | BP | 0.000667 |
| GO:0045766 | positive regulation of angiogenesis | BP | 0.000747 |
| GO:0004175 | endopeptidase activity | MF | 0.000748 |
| GO:0098631 | cell adhesion mediator activity | MF | 0.0008 |
| GO:0040017 | positive regulation of locomotion | BP | 0.000971 |
| GO:1904018 | positive regulation of vasculature development | BP | 0.000971 |
| GO:0008528 | G-protein coupled peptide receptor activity | MF | 0.000992 |
| GO:0004222 | metalloendopeptidase activity | MF | 0.000992 |
| GO:0001653 | peptide receptor activity | MF | 0.000992 |
| GO:0004930 | G-protein coupled receptor activity | MF | 0.000997 |
| GO:0008236 | serine-type peptidase activity | MF | 0.000997 |
| GO:0030545 | receptor regulator activity | MF | 0.000997 |
| GO:0030414 | peptidase inhibitor activity | MF | 0.001256 |
| GO:0017171 | serine hydrolase activity | MF | 0.001595 |
| GO:0002685 | regulation of leukocyte migration | BP | 0.001942 |
| GO:0006937 | regulation of muscle contraction | BP | 0.002032 |
| GO:0001655 | urogenital system development | BP | 0.002061 |
| GO:0005865 | striated muscle thin filament | CC | 0.002126 |
| GO:0000778 | condensed nuclear chromosome kinetochore | CC | 0.002156 |
| GO:0006936 | muscle contraction | BP | 0.002169 |
| GO:0003158 | endothelium development | BP | 0.002257 |
