# Supplementary material for: 3D spheroids of human placenta-derived mesenchymal stem cells attenuate spinal cord injury in mice
Source: Cell Death Dis. 2021 Nov 22;12(12):1096. doi: 10.1038/s41419-021-04398-w (PMC8606575; doi:10.1038/s41419-021-04398-w)
Supplement: Supplementary file 11 — Sup. table 5 [file 41419_2021_4398_MOESM11_ESM.docx]

Sup. table 5 List of the top 20 terms of KEGG pathway following 3D-spheroid culture

| Pathway | 3D number | Control number | P value adjust |
| --- | --- | --- | --- |
| Neuroactive ligand-receptor interaction | 32 | 136 | 0.001288 |
| Cell cycle | 27 | 119 | 0.007216 |
| Cytokine-cytokine receptor interaction | 30 | 167 | 0.142261 |
| Complement and coagulation cascades | 13 | 53 | 0.156403 |
| ECM-receptor interaction | 15 | 68 | 0.181067 |
| Terpenoid backbone biosynthesis | 7 | 21 | 0.181067 |
| Hematopoietic cell lineage | 11 | 46 | 0.23328 |
| Pathogenic Escherichia coli infection | 11 | 47 | 0.23328 |
| Steroid biosynthesis | 6 | 18 | 0.23328 |
| PI3K-Akt signaling pathway | 41 | 277 | 0.271721 |
| Axon guidance | 25 | 152 | 0.2722 |
| Protein digestion and absorption | 13 | 64 | 0.2722 |
| MicroRNAs in cancer | 25 | 153 | 0.2722 |
| Pathways in cancer | 59 | 438 | 0.310691 |
| Taste transduction | 8 | 33 | 0.310691 |
| p53 signaling pathway | 13 | 67 | 0.310691 |
| DNA replication | 8 | 34 | 0.313564 |
| Arrhythmogenic right ventricular cardio-myopathy | 12 | 61 | 0.313564 |
| Calcium signaling pathway | 22 | 141 | 0.447198 |
| Cholesterol metabolism | 8 | 38 | 0.510073 |
