# Supplementary material for: 3D spheroids of human placenta-derived mesenchymal stem cells attenuate spinal cord injury in mice
Source: Cell Death Dis. 2021 Nov 22;12(12):1096. doi: 10.1038/s41419-021-04398-w (PMC8606575; doi:10.1038/s41419-021-04398-w)
Supplement: Supplementary file 16 — Author Contribution Statement [file 41419_2021_4398_MOESM16_ESM.docx]

**Author contribution statement**

Junhao Deng wrote this initial manuscript. Junhao Deng, and Miao Li equally performed all the experiments and analyzed all of the relative data. Fanqi Meng, Zhongyang Liu, Song Wang, Yuan Zhang, and Ming Li participated in analyzing some of the relevant data. Zhirui Li, Licheng Zhang and Peifu Tang instructed all the process of this study. All authors read and approved the final manuscript.
